# Two new hermit crab species of *Diogenes* (Crustacea: Decapoda: Diogenidae) from Atlanto‐Mediterranean coasts of Iberian Peninsula: Poleward migrants or merely overlooked indigenous species?

**DOI:** 10.1002/ece3.8844

**Published:** 2022-05-19

**Authors:** Bruno Almón, Jose A. Cuesta, J. Enrique García‐Raso

**Affiliations:** ^1^ Centro Oceanográfico de Vigo IEO‐CSIC Vigo Spain; ^2^ Grupo de Estudo do Medio Mariño (GEMM) A Coruña Spain; ^3^ Instituto de Ciencias Marinas de Andalucía ICMAN‐CSIC Cádiz Spain; ^4^ Facultad de Ciencias Departamento de Biología Animal Universidad de Málaga Málaga Spain

**Keywords:** anomura, biodiversity, biogeography, molecular systematics, species delineation, taxonomy, tropicalization

## Abstract

A new hermit crab species of the genus *Diogenes* with reddish‐orange cheliped, *Diogenes erythromanus* sp. nov., is described and illustrated based on specimens from the Mediterranean coasts of the Iberian Peninsula, southern Spain. In addition, a second morphotype originating from Mauritanian waters and morphologically very close to *D. erythromanus* sp. nov. is described as a different species, *D. arguinensis* sp. nov. The new species are here compared to morphologically similar congeners, especially to those inhabiting the same geographical range. *Diogenes erythromanus* sp. nov. is distinguishable from other *Diogenes* primarily by the shape and armature of the left cheliped, with a palm slightly higher than long, with a ridge of spines running along the proximal lower margin that continues with a series of spinose rows forming a central band parallel to the upper margin of the palm. The palm in *D. arguinensis* sp. nov. is longer than high and shows similar proximal ridge, but without central spinose ridge. The shape of the cheliped is also different in *D. arguinensis* sp. nov., with long dactylus, which is also flattened and twisted. Sequences from two mitochondrial and one nuclear genes, and comparative analyses with other available sequences for the genus, are also included. Molecular phylogenetic analyses support the morphological delimitation, with *D. erythromanus* sp. nov. and *D. arguinensis* sp. nov. forming a separate group, more related to other tropical species, which raises different possible explanations for its presence in the Iberian Peninsula.

## Graphical abstract

Two new species of *Diogenes* with presence in both European and African waters, previously included under the name *Diogenes pugilator*, are described under the light of the modern integrative taxonomy.

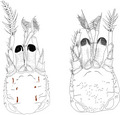

## 1 INTRODUCTION

The hermit crab genus *Diogenes* Dana, 1851, is currently represented by 74 species worldwide (Almón et al., 2021; Komai & Yoshida, 2020; Lemaitre & McLaughlin, 2022), with the number of new species being described in continuous increase in recent years, as a consequence of the implementation and generalization of molecular tools, which has allowed to address ancient taxonomic dilemmas and clarify species identities. Most of the efforts made in the study of this genus has been focused on Indo‐West Pacific coasts, with several new species described in the last three decades (Asakura, 2006, 2020; Asakura & Godwin, 2006; Asakura & Tachikawa, 2010; Igawa & Kato, 2017; Komai et al., 2012, 2013, 2018; Komai & Yoshida, 2020; McLaughlin & Clark, 1997; McLaughlin & Holthuis, 2001; Morgan & Forest, 1991; Rahayu, 1996, 2012, 2015; Rahayu & Forest, 1995; Rahayu & Hortle, 2002; Siddiqui & McLaughlin, 2003; Trivedi et al., 2016; Xiao et al., 2015). However, the study of the diogenids in the Atlantic had its peak five decades ago and has remained since then scarce and outdated (Almón et al., 2021; Landschoff & Rahayu, 2018).

The recent revision and comparison of specimens belonging to the genus *Diogenes* originating from the coasts of the Iberian Peninsula, and nearby areas, revealed the existence of a species complex so far included in the nominal species *Diogenes pugilator* (Roux, 1829). The study of these material led to the redescription of *Diogenes pugilator*, the resurrection of *D*. *curvimanus* (Clément, 1874), an ancient synonym, and the description of *D*. *armatus* Almón et al., 2021 as a new species (Almón et al., 2021), adding two species to the eight already known from the Atlantic. In that work, it was stated that several other morphotypes were identified as different and should be addressed in a separate work. Some of them were included in the phylogenetic analysis and the results of the phylogeny showed them as valid species and distinguishable from the rest, but without matching any of species known to date.

The present work deals with two of these morphotypes, preliminary labeled as *Diogenes* sp1 and *Diogenes* sp2, and has as main objectives: (1) to describe both morphotypes as new species for the genus, (2) to update the information about the genus within European waters, (3) investigate their possible origin and inquire whether their arrival in Europe might be the result of recent migration or otherwise, and (4) to update the identification key for the Atlantic diogenid species.

## 2 MATERIALS AND METHODS

Specimens included in this study come from different sources and were obtained during sampling trips conducted during the period 2018–2020. Additional samples were recovered from the crustacean reference collection “Colección de Crustáceos Decápodos y Estomatópodos del Centro Oceanográfico de Cádiz (CCDE‐IEOCD),” belonging to the Spanish Institute of Oceanography (IEO‐CSIC; Muñoz & García‐Isarch, 2021), and from Biological Reference Collections (CBR) at the Institut de Ciències del Mar (ICM‐CSIC, Barcelona, Spain; Guerrero et al., 2020), to complete the information about the distribution of these species and to explore the hypothesis of their possible recent arrival to Iberian waters. All specimens were studied under the stereomicroscope and classified to the lowest taxonomical level possible.

For preventing damage of key structures for morphological identification, a piece of one of the antennae or single ambulatory leg were used as tissue sample for DNA extraction in males and non‐ovigerous females, while eggs were employed for ovigerous females. Extraction protocol follows that from Estoup et al. (1996) and was carried out at the Instituto de Ciencias Marinas de Andalucía (ICMAN‐CSIC). Partial gene sequences were amplified using the following PCR thermal cycles: initial denaturing for 5 min at 95°C; followed by 40 cycles: 30 s at 95°C, 30 s at 45–56°C (depending on primer pairs, see Table 1), 45 s at 72°C; and a final extension of 3 min at 72°C. New specific COI primers (DiogF and DiogR) were designed for this study due to difficulties in obtaining PCR amplification with the commonly used universal primers. PCR amplicons for partial sequences of the 16S, COI, and 28S genes were sent for purification and sequencing to external laboratories (Stab Vida).

**TABLE 1:** List of primer sequences used in this study for the PCR amplification of partial sequences of 16S, COI and 28S genes, including pair combined, annealing temperature for each primer pair (AT), length of the sequences obtained (bp), and references

Gene	Primer	Sequence	Pair	AT (°C)	bp	Reference
16S	1472	5′‐AGA TAG AAA CCA ACC TGG‐3′	16L2	45	570	Crandall and Fitzpatrick (1996)
	16L2	5′‐TGC CTG TTT ATC AAA AAC AT‐3′				Schubart et al. (2002)
	16br	5′‐CCG GTC TGA ACT CAG ATC ACG T‐3′	16L12	52	450	Palumbi et al. (1991)
	16L12	5′‐TGA CCG TGC AAA GGT AGG ATA A‐3′				Schubart et al. (1998)
						
COI	DiogF	5′‐TTG GWG CWT GRG CYG GWA TAG‐3′	DiogR / COH6	54 / 54	580/625	Present study
	DiogR	5′‐GGA TCY CCW CCW CCW GCH GGA‐3′				Present study
	COH6	5′‐TAD ACT TCD GGR TGD CCA AAR AAY CA‐3′	COL6b	45	670	Schubart and Huber (2006)
	COL6b	5′‐ACA AAT CAT AAA GAT ATY GG‐3′				Schubart and Huber (2006)
						
28S	28L1	5′‐CGG AGG AAA AGA AAC CAA CAG‐3′	28DH2	56	750	Mock and Schubart (2021)
	28D2H	5′‐TGA CTC GCA CAC ATG TTA GA‐3′			750	Mock and Schubart (2021)

Consensus sequences were generated from the complementary sequences with Bioedit vr. 7.0.5 (Hall, 1999). BLAST searches were performed for each of these sequences in GenBank and for the COI also in BoLD to compare with the available information, confirm or refute the identification, and detect possible issues in these databases.

For the phylogenetic approach, 31 of the available sequences belonging to *Diogenes* were downloaded from the National Center for Biotechnology Information (NCBI) database (Table 2) and assembled along with the 153 sequences generated in a previous study (Almón et al., 2021) and the 30 new sequences generated in this study. The final dataset was then aligned by MUltiple Sequence Comparison by Log‐Expectation (MUSCLE; Edgar, 2004), implemented in MEGA X version 10.2 (Kumar et al., 2018).

**TABLE 2:** List of DNA sequences of *Diogenes* included in the present study, including newly generated and retrieved from previous study, along with the selected sequences downloaded from NCBI/BOLD databases, with voucher numbers, collection area and GenBank/BoLD accession codes for 16S, COI, and 28S partial sequences; type specimens are indicated by an asterisk and sequences generated in this study are shown in bold

Species	Collection location	Voucher	Gene		
			16S	COI	28S
*Diogenes curvimanus*	Spain	MNHN‐IU−2019–3214*	MW791779	MW776663	MW802642
*Diogenes curvimanus*	Spain	IEOCD‐BR/2581	MW791781	MW776675	‐
*Diogenes curvimanus*	Spain	IEOCD‐BR/2582	MW791782	MW776662	MW802643
*Diogenes curvimanus*	Spain	ZSMA2019 0398	MW791784	MW776672	‐
*Diogenes curvimanus*	Spain	IEOCD‐BR/2596	MW791785	MW776674	‐
*Diogenes curvimanus*	Spain	IEOCD‐BR/2597	MW791786	MW776673	‐
*Diogenes curvimanus*	Spain	IEOCD‐BR/2598	MW791792	MW776669	‐
*Diogenes curvimanus*	Spain	IEOCD‐BR/2599	MW791788	MW776668	MW802644
*Diogenes curvimanus*	Spain	IEOCD‐BR/2600	MW791789	MW776667	‐
*Diogenes curvimanus*	Spain	IEOCD‐BR/2601	MW791787	MW776676	‐
*Diogenes curvimanus*	Spain	IEOCD‐BR/2604	MW791783	‐	‐
*Diogenes curvimanus*	Spain	IEOCD‐BR/2605	MW791790	MW776671	MW802645
*Diogenes curvimanus*	Spain	IEOCD‐BR/2606	MW791791	MW776670	MW802646
*Diogenes curvimanus*	Spain	IEOCD‐BR/2607	MW791777	MW776666	MW802639
*Diogenes curvimanus*	Spain	IEOCD‐BR/2608	MW791778	MW776665	MW802640
*Diogenes curvimanus*	Spain	IEOCD‐BR/2609	MW791780	MW776664	MW802641
*Diogenes curvimanus*	Belgium	IEOCD‐BR/2612	‐	MW776659	‐
*Diogenes curvimanus*	Belgium	IEOCD‐BR/2618	‐	MW776658	MW802648
*Diogenes curvimanus*	Belgium	IEOCD‐BR/2619	‐	MW776660	‐
*Diogenes curvimanus*	France	IEOCD‐BR/2621	MW791793	MW776661	MW802647
*Diogenes curvimanus*	Spain	IEOCD‐BR/2622	MW791776	‐	‐
*Diogenes armatus*	Spain	MNHN‐IU−2014–5736*	MW791814	MW776705	MW802658
*Diogenes armatus*	Spain	MNHN‐IU−2019–3213*	MW791815	MW776704	MW802659
*Diogenes armatus*	Spain	IEOCD‐BR/2645	MW791813	MW776701	MW802657
*Diogenes armatus*	Spain	ZSMA2019 0402	MW791806	MW776709	MW802653
*Diogenes armatus*	Spain	IEOCD‐BR/2623	MW791818	MW776700	‐
*Diogenes armatus*	Spain	IEOCD‐BR/2624	MW791820	MW776695	‐
*Diogenes armatus*	Spain	IEOCD‐BR/2625	MW791810	MW776696	‐
*Diogenes armatus*	France	IEOCD‐BR/2627	‐	MW776697	MW802661
*Diogenes armatus*	Corsica	IEOCD‐BR/2628	MW791816	MW776699	‐
*Diogenes armatus*	Spain	IEOCD‐BR/2631	‐	MW776698	‐
*Diogenes armatus*	Spain	IEOCD‐BR/2639	‐	‐	MW802660
*Diogenes armatus*	Tunisia	IEOCD‐BR/2640	MW791817	‐	‐
*Diogenes armatus*	Tunisia	IEOCD‐BR/2641	MW791819	‐	‐
*Diogenes armatus*	Spain	IEOCD‐BR/2642	MW791807	MW776708	MW802654
*Diogenes armatus*	Spain	IEOCD‐BR/2643	MW791811	MW776703	MW802656
*Diogenes armatus*	Spain	IEOCD‐BR/2644	MW791812	MW776702	‐
*Diogenes armatus*	Portugal	IEOCD‐BR/2647	MW791808	MW776707	MW802655
*Diogenes armatus*	Portugal	IEOCD‐BR/2648	MW791809	MW776706	
*Diogenes pugilator*	France	MNHN‐IU−2019–3215*	‐	MW776683	‐
*Diogenes pugilator*	Tunisia	IEOCD‐BR/2659	MW791795	‐	‐
*Diogenes pugilator*	Spain	IEOCD‐BR/2660	MW791796	MW776692	**OM523062**
*Diogenes pugilator*	Spain	IEOCD‐BR/2661	MW791797	MW776688	**OM523063**
*Diogenes pugilator*	France	ZSMA2019 0400	‐	MW776678	‐
*Diogenes pugilator*	France	ZSMA2019 0401	‐	MW776681	‐
*Diogenes pugilator*	Spain	ICMD 143/1998a	MW791805	MW776686	‐
*Diogenes pugilator*	Spain	ICMD 143/1998b	‐	MW776687	‐
*Diogenes pugilator*	Spain	IEOCD‐BR/2662	MW791801	MW776694	‐
*Diogenes pugilator*	France	IEOCD‐BR/2664	MW791804	MW776677	‐
*Diogenes pugilator*	France	IEOCD‐BR/2665	‐	MW776684	‐
*Diogenes pugilator*	France	IEOCD‐BR/2666	‐	MW776682	‐
*Diogenes pugilator*	France	IEOCD‐BR/2667	‐	MW776685	‐
*Diogenes pugilator*	France	IEOCD‐BR/2668	‐	MW776680	‐
*Diogenes pugilator*	France	IEOCD‐BR/2669	‐	MW776679	‐
*Diogenes pugilator*	Spain	IEOCD‐BR/2670	MW791802	‐	‐
*Diogenes pugilator*	Tunisia	IEOCD‐BR/2673	MW802638	‐	‐
*Diogenes pugilator*	Spain	IEOCD‐BR/2674	MW791799	MW776690	‐
*Diogenes pugilator*	Spain	IEOCD‐BR/2675	MW791800	MW776689	‐
*Diogenes pugilator*	France	IEOCD‐BR/2676	‐	‐	MW802652
*Diogenes pugilator*	Spain	IEOCD‐BR/2677	MW791798	MW776691	‐
*Diogenes pugilator*	Spain	IEOCD‐BR/2678	MW791803	MW776693	‐
*Diogenes ovatus*	Mauritania	IEO‐CD‐CCLME11/1572‐1	MW791794	**OM523188**	
*Diogenes ovatus*	Guinea Conakry	IEO‐CD‐CCLME11/1667	‐	‐	MW802650
*Diogenes ovatus*	Guinea‐Bissau	IEO‐CD‐CCLME12/2569	**OM523035**	MW776721	MW802649
*Diogenes ovatus*	Guinea‐Bissau	IEO‐CD‐CCLME12/2571	**OM523036**	MW776720	MW802651
***Diogenes arguinensis* sp. nov**.	Spain	IEOCD‐BR/2682	MW791826	MW776713	**OM523064**
***Diogenes arguinensis* sp. nov**.	Spain	IEOCD‐BR/2683	MW791825	MW776712	‐
***Diogenes arguinensis* sp. nov**.	Spain	IEOCD‐BR/2684	MW791827	MW776718	‐
***Diogenes arguinensis* sp. nov**.	Mauritania	IEO‐CD‐CCLME12/2572*	MW791830	MW776715	‐
***Diogenes arguinensis* sp. nov**.	Mauritania	IEO‐CD‐CCLME12/2573	MW791824	‐	‐
***Diogenes arguinensis* sp. nov**.	Mauritania	IEO‐CD‐CCLME12/2575	MW791831	MW776714	‐
***Diogenes arguinensis* sp. nov**.	Morocco	IEO‐CD‐CCLME12/2576‐1	MW791823	MW776719	MW802664
***Diogenes arguinensis* sp. nov**.	Morocco	IEO‐CD‐CCLME12/2577‐1	MW791829	MW776716	‐
***Diogenes arguinensis* sp. nov**.	Morocco	IEO‐CD‐CCLME11/690‐1	**OM523037**	MW776717	‐
***Diogenes arguinensis* sp. nov**.	Spain	IEOCD‐BR−2918	**OM523038**	**OM523183**	**OM523065**
***Diogenes arguinensis* sp. nov**.	Spain	IEOCD‐BR−2919	**OM523039**	‐	‐
***Diogenes arguinensis* sp. nov**.	Spain	IEOCD‐BR−2920	**OM523040**	‐	‐
***Diogenes arguinensis* sp. nov**.	Spain	IEOCD‐BR−2922	**OM523041**	**OM523184**	**OM523066**
***Diogenes arguinensis* sp. nov**.	Spain	IEOCD‐BR−2923	**OM523042**	‐	‐
***Diogenes arguinensis* sp. nov**.	Spain	IEOCD‐BR−2924	**OM523043**	‐	‐
***Diogenes arguinensis* sp. nov**.	Spain	IEOCD‐BR−2925	**OM523044**	**OM523185**	‐
***Diogenes arguinensis* sp. nov**.	Spain	IEOCD‐BR−2926	**OM523045**	‐	‐
***Diogenes arguinensis* sp. nov**.	Spain	IEOCD‐BR−2927	**OM523046**	**OM523186**	‐
***Diogenes arguinensis* sp. nov**.	Spain	IEOCD‐BR−2928	**OM523047**	‐	‐
***Diogenes arguinensis* sp. nov**.	Spain	IEOCD‐BR−2929	**OM523048**	**OM523187**	‐
***Diogenes arguinensis* sp. nov**.	Spain	IEOCD‐BR−2930	**OM523049**	‐	‐
***Diogenes arguinensis* sp. nov**.	Spain	IEOCD‐BR−2931	**OM523050**	‐	‐
***Diogenes arguinensis* sp. nov**.	Spain	IEOCD‐BR−2932	**OM523051**	‐	‐
***Diogenes erythromanus* sp. nov**.	Spain	IEOCD‐BR/2680*	MW791821	MW776710	MW802662
***Diogenes erythromanus* sp. nov**.	Morocco	IEO‐CD‐CCLME12/2578‐1	MW791822	MW776711	MW802663
***Diogenes erythromanus* sp. nov**.	Spain	IEOCD‐BR−2921	**OM523052**		
***Diogenes erythromanus* sp. nov**.	Spain	IEOCD‐BR−2933	**OM523053**		
*Diogenes albimanus*	South Africa	MB‐A066353	‐	MH482073	‐
*Diogenes pugilator*	North Sea	‐	‐	BNSC192‐11	‐
*Diogenes pugilator*	North Sea, German Bight	‐	‐	BNSDE084‐11	‐
*Diogenes pugilator*	North Sea, German Bight	‐	‐	BNSDE086‐11	‐
*Diogenes miles*	India	‐	‐	GBCMA6701‐14	‐
*Diogenes alias*	India	‐	‐	GBCMA6707‐14	‐
*Diogenes canaliculatus*	India	‐	‐	GBCMA6708‐14	‐
*Diogenes dubius*	India	‐	‐	GBCMA6709‐14	‐
*Diogenes manaarensis*	India	‐	‐	GBCMA6710‐14	‐
*Diogenes merguiensis*	India	‐	‐	GBCMA6711‐14	‐
*Diogenes planimanus*	India	‐	‐	GBCMA6717‐14	‐
*Diogenes violaceus*	India	‐	‐	GBCMA6718‐14	‐
*Diogenes brevirostris*	South Africa, Western Cape	‐	‐	HONS017‐19	‐
*Diogenes brevirostris*	South Africa	HVDBC−53	‐	HVDBC053‐11	‐
*Diogenes viridis*	Vanuatu	MNHN‐IU−2008–16281	‐	MDECA648‐10	‐
*Diogenes viridis*	Vanuatu	MNHN‐IU−2008–16282	‐	MDECA649‐10	‐
*Diogenes pallescens*	Vanuatu	MNHN‐IU−2008–16294	‐	MDECA658‐10	‐
*Diogenes pallescens*	Vanuatu	MNHN‐IU−2008–16297	‐	MDECA660‐10	‐
*Diogenes pugilator*	Portugal, Alentejo	LMBSWB1‐001	‐	MLALE067‐14	‐
*Diogenes pugilator*	Portugal, Alentejo	LMBSWB1‐002	‐	MLALE068‐14	‐
*Diogenes pugilator*	Portugal, Alentejo	LMBSWB1‐003	‐	MLALE069‐14	‐
*Diogenes costatus*	South Africa: KwaZulu‐Natal	MB‐A066693	‐	MH481985	‐
*Diogenes costatus*	South Africa: Western Cape	MB‐A066759	‐	MH481993	‐
*Diogenes spinicarpus*	Vanuatu	MNHN‐IU−2008–16275	‐	MDECA642‐10	‐
*Diogenes spinicarpus*	Vanuatu	MNHN‐IU−2008–16276	‐	MDECA643‐10	‐
*Diogenes goniochirus*	China	‐	MK610031	‐	‐
*Diogenes edwardsii*	China	‐	MK610030	‐	‐
*Diogenes nitidimanus*	China	‐	MK610029	‐	‐
*Diogenes rectimanus*	China	‐	MK610028	‐	‐
*Diogenes deflectomanus*	China	‐	MK610027	‐	‐
*Diogenes avarus*	China	‐	MK610026	‐	‐
*Paguristes eremita*	Morocco	IEO‐CD‐CCLME11/1591	MW791833	MW776657	‐
*Dardanus arrosor*	Morocco	IEO‐CD‐CCLME11/1575	MW791834	MW776656	‐

Blocks of ambiguous data in the non‐protein‐coding gene alignments were identified and excluded using Gblocks with relaxed settings (Talavera & Castresana, 2007). Gene concatenation of the COI +16S (1187 bp) and the best‐fitting nucleotide substitution models for each gene and for the entire alignment were assessed with the tools implemented in MEGA X, using the corrected Akaike information criterion, as recommended by Posada and Buckley (2004). According to the results of this method, the Tamura 3‐parameter model of nucleotide substitution using discrete gamma‐distributed rates for the variable sites and with invariant sites (T92+G+I) was selected in all cases. Estimates of evolutionary divergence between sequences were obtained using the pairwise distances calculation tool implemented in MEGA X.

Maximum‐likelihood (ML) analyses were conducted for the concatenated dataset, as well as for the individual genes (16S, COI, and 28S). Concatenated analyses were partitioned based on gene identity (i.e., 16S and COI). Two species of the family Diogenidae, *Dardanus arrosor* (Herbst, 1796) and *Paguristes eremita* (Linnaeus, 1767), were selected as outgroups. ML analyses were performed using MEGA X software under the T92+G+I model. A random starting tree was generated using the Neighbor‐Joining method, selecting the partial deletion option (75% site coverage cutoff). A ML tree was generated using the Nearest‐Neighbor interchange option. Topological robustness was investigated using 1000 nonparametric bootstrap replicates. In the resulting trees, only the values >70% nodal support are shown.

## 3 RESULTS

### 3.1 Systematic account

#### 3.1.1 Family Diogenidae Ortmann, 1892

**Genus** ***Diogenes* Dana, 1851**.

**FIGURE 1:**
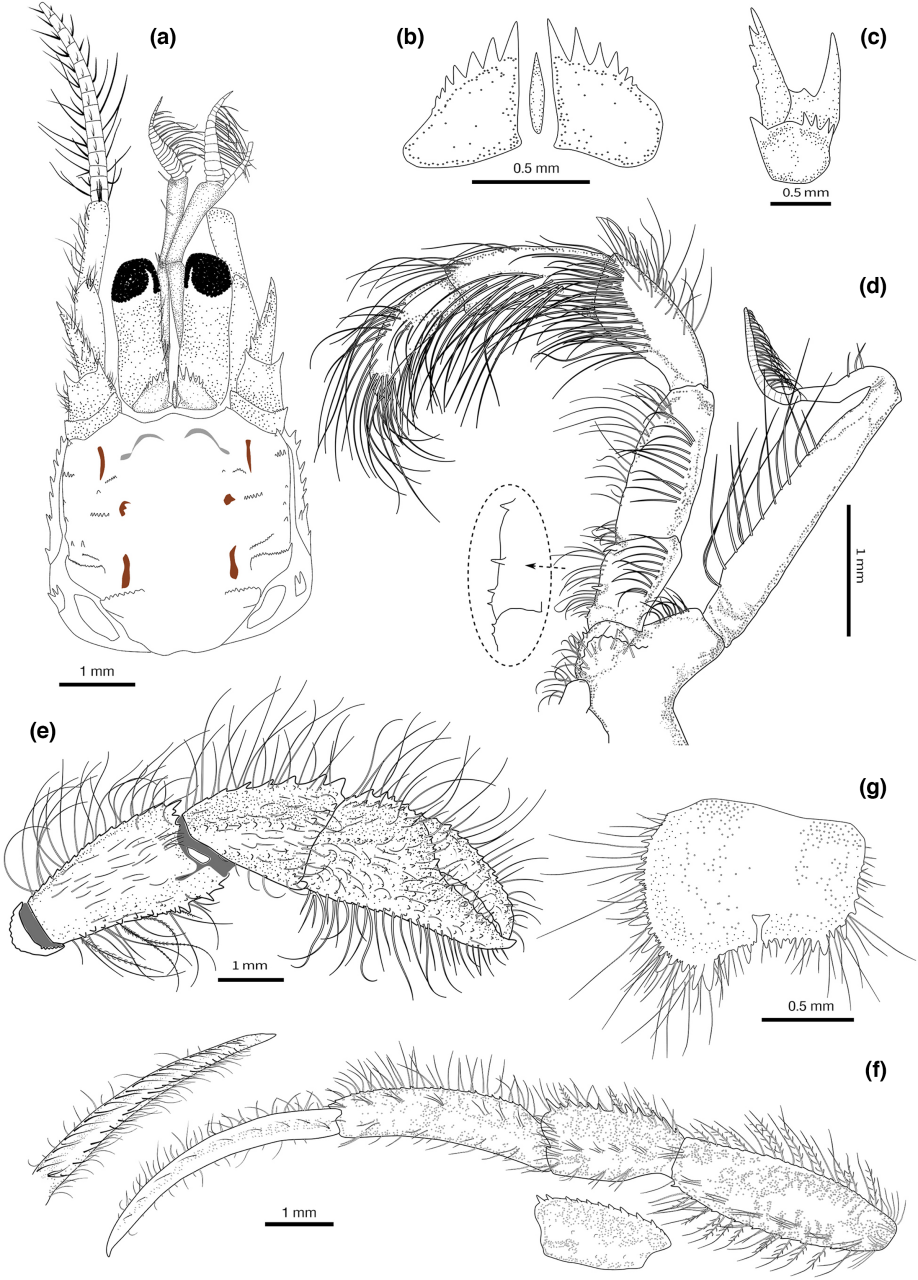
*Diogenes erythromanus* sp. nov. **Málaga**, ♀ 2.8 mm, Spain, holotype, (IEOCD‐BR/2680): (a) Anterior part of carapace and cephalic appendages, dorsal view; (b) ocular acicles and intercalary rostriform process, dorsal view; (c) right antennal peduncle, mesial face; (d) left maxiliped 3 (inset: detail of the spines, ventral aspect); (g) Telson, dorsal view. *Diogenes erythromanus* sp. nov. **Morocco**, ♂ 3.0 mm, paratype (IEO‐CD‐CCLME12/2578‐2): (e) right cheliped, outer view; (f) Left pereiopod 2 (insets—variations in P3 carpus; dactylus inner view). (Scales: a, e‐g = 1 mm; b‐c, g = 0.5 mm)

**FIGURE 2:**
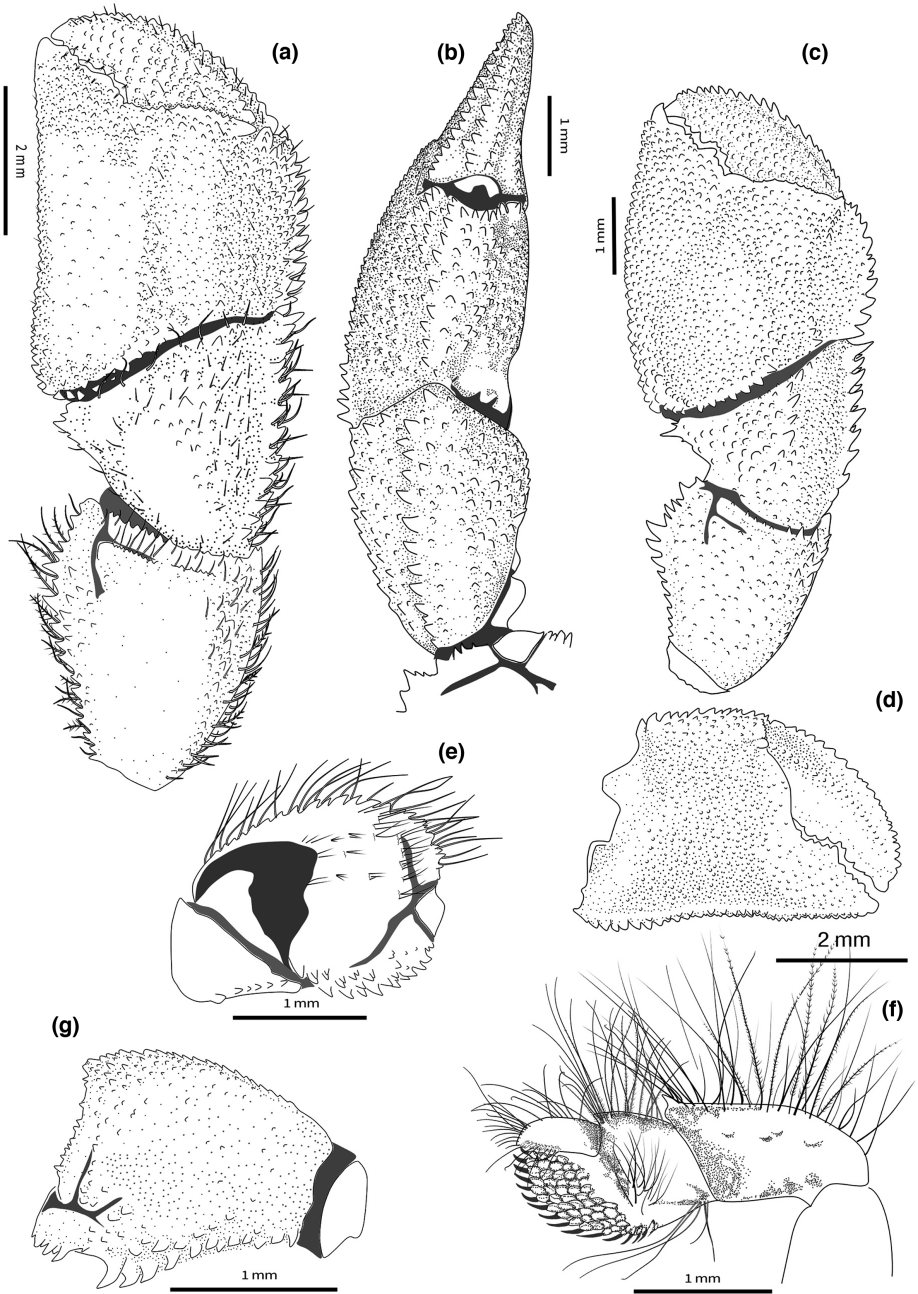
*Diogenes erythromanus* sp. nov. **Morocco**, ♂ 3.0 mm, paratype, (IEO‐CD‐CCLME12/2578‐2): (a) Left cheliped, outer view; (b) left cheliped, dorsal view; (d) left cheliped, palm inner surface; (e) merus, mesial view (seat omitted); (g) merus, outer view. *Diogenes erythromanus* sp. nov. **Málaga**, Spain, ♀ 2.8 mm, holotype, (IEOCD‐BR/2680): (c) Left cheliped, outer view. (f) pereiopod 4. (Scales: a, d = 2 mm; b‐c, e‐g = 1 mm)

**FIGURE 3:**
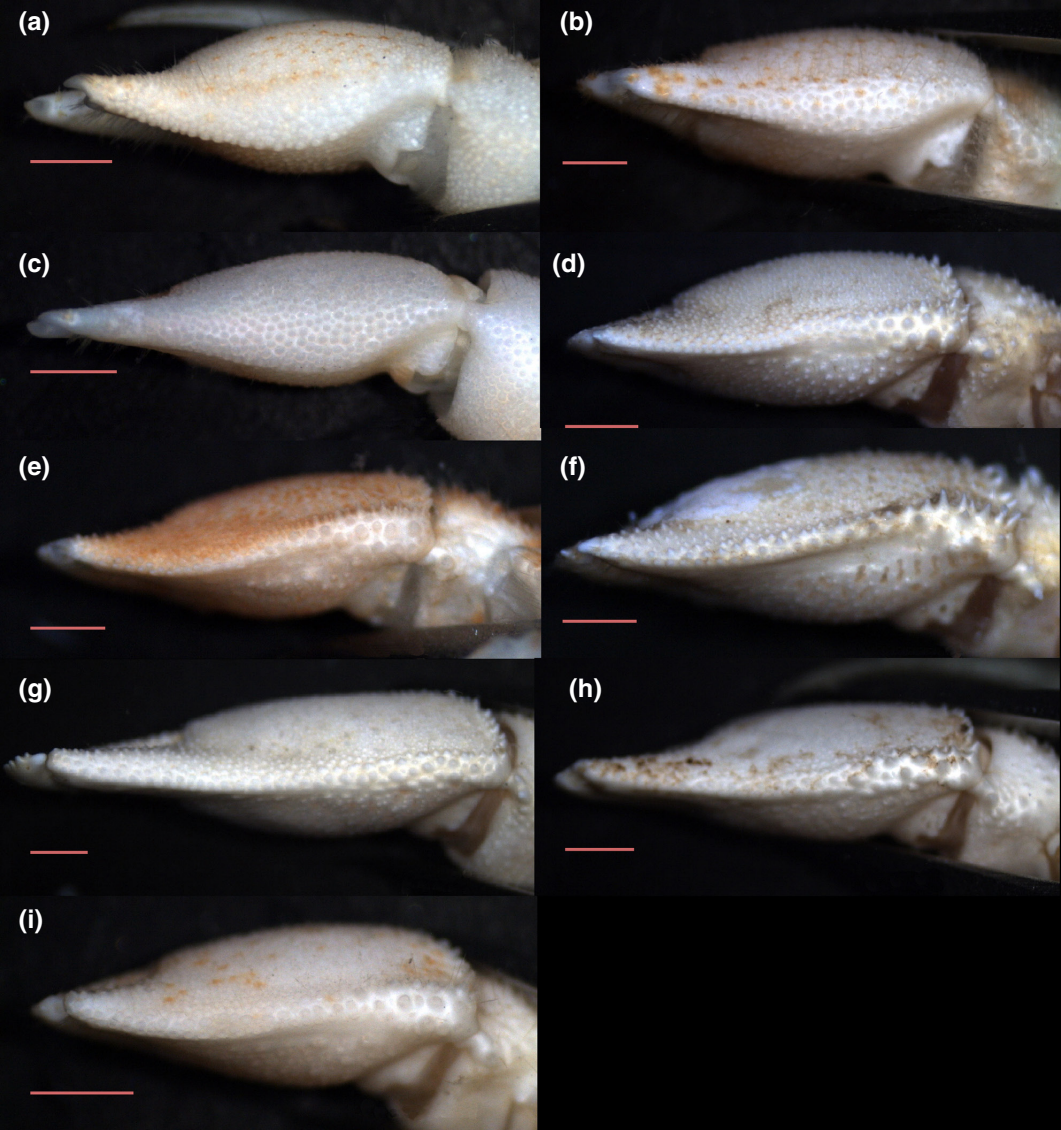
a‐i, palm of the left cheliped, lower margin in ventral view: *Diogenes pugilator* (a) ♂, topotype (IEOCD‐BR/2667); *Diogenes armatus* (b) ♂, holotype (MNHN‐IU‐2019–3213); *Diogenes curvimanus* (c) ♂, topotype (IEOCD‐BR/2581); *Diogenes erythromanus* sp. nov. (d) ♂, paratype (IEOCD‐CCLME12/2578‐2), (e) ♀ holotype (IEOCD‐BR/2680), (f) ♀ paratype (IEOCD‐CCLME12/2578‐1); *Diogenes arguinensis* sp. nov. (g) ♂ holotype (IEO‐CD‐CCLME12/2572), (h) ♂ paratype (IEO‐CD‐CCLME12/2575), (i) ♀ (IEO‐CD‐CCLME12/2576‐1). (Scales: a‐f = 1 mm)

**FIGURE 4:**
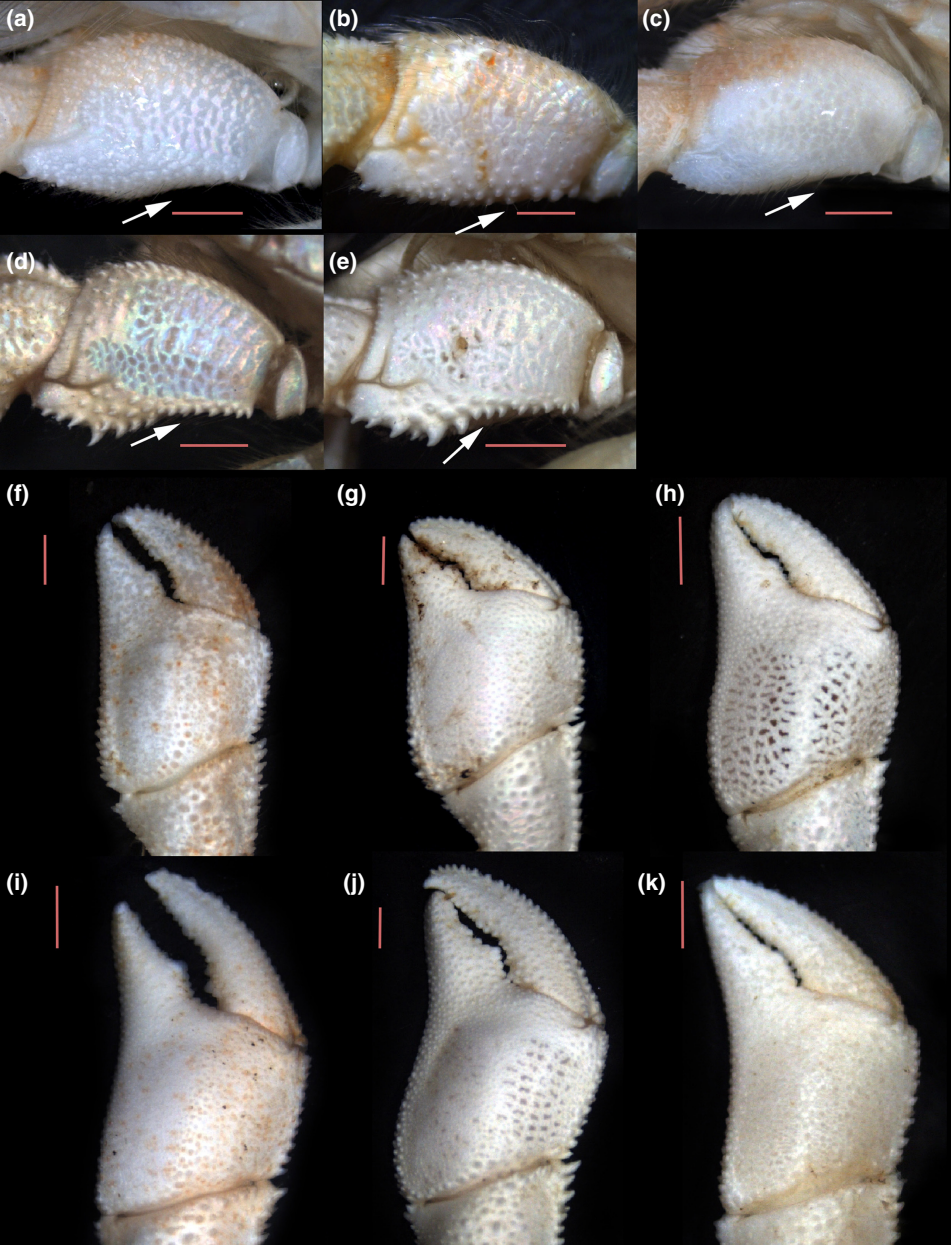
a–d, male left cheliped merus of the *Diogenes* species present in the Iberian Peninsula, outer view (arrow indicating ventrolateral outer margin): *Diogenes pugilator* (a) ♂, topotype (IEOCD‐BR/2667); *Diogenes armatus* (b) ♂, holotype (MNHN‐IU‐2019–3213); *Diogenes curvimanus* (c) ♂, topotype (IEOCD‐BR/2581); *Diogenes erythromanus* sp. nov. (d) ♂, paratype (IEOCD‐CCLME12/2578‐2); *Diogenes arguinensis* sp. nov. (e) ♂ paratype (IEO‐CD‐CCLME12/2575). f‐k, variations in palm of left cheliped in males of *Diogenes arguinensis* sp. nov.; (f, i) Spain; (g, h, j) Mauritania; (k) Morocco

*Diogenes erythromanus* sp. nov.

(Figures 1a‐g, 2a‐g, 3d‐f, 4d).

LSID:zoobank.org:act:50067F29‐4086‐41C7‐8714‐DCE0F3FC5326.

**Holotype:** 1♀ 2.8 mm, (IEOCD‐BR/2680), **Spain**: Baños del Carmen, Málaga, 36°43'02.4"N, 4°23'12.6"W, sand, subtidal, 15 m, June 14, 2018.

**Paratypes:** 1♀ 3.2 mm, 1♂ 3.0 mm (IEO‐CD‐CCLME12‐2578–1, 2578–2), **Morocco**: off Kenitra, 34°23'16.8"N, 6°37'27.5"W sand, subtidal, 31 m, July 11, 2012.

Other material: **Spain**: 1♂, 1♀ (IEO‐CD‐BR‐2921, 2933), Cabo Pino, Mijas, Málaga, 36⁰29’42’’N, 4⁰39’36’’W fine sand, subtidal, 15 m, September 23, 2009; **Morocco**: 2♂, 6♀ (IEO‐CD‐CCLME12/2578‐3), same data as Paratype.

**Description: Shield** (Figure 1a) subquadrate, slightly longer than broad, not vaulted; rostral lobe broadly rounded, exceeded by lateral projections, which are triangular, acutely pointed, with single spine at apex; anterior margins between rostral lobe and lateral projections slightly concave; anterolateral margins sloping, smooth; anterolateral angles rounded, usually with one terminal and one subterminal spines; lateral margins almost straight, with posterior halves each cut by two transverse, spinulose ridges on proximal half, extending onto lateral surface of shield; posterior margin truncate; dorsal surface with additional subtle short transverse rows of small tubercles and tufts of short stiff setae; additional longitudinal row of 4–5 small spines adjacent to lateral margins in distal half. Branchiostegites with row of 5 + 1 paired +1 strong spines on dorsal margin. Posterolateral plates not well calcified, unarmed.

**Ocular peduncles** (including corneas) about 0.5–0.6 times as long as shield, moderately stout, slightly narrowed medially; corneas not dilated, corneal diameter about 0.3 peduncle length; row of short plumose setae on mesial margin of peduncles. **Ocular acicles** (Figure 1a,b) subtriangular, with sinuous mesial margin; anterior margin slightly convex, bearing 4–5 large acute distal spines and 2–3 small spines (sometimes reduced to small tubercles) decreasing in size toward outer margin; innermost 2 distal spines distinctly larger; rest of latero‐anterior outer margin smooth, occupying about 1/3 of anterior margin. **Intercalary rostriform process** simple, slender, slightly shorter than or subequal in length to ocular acicles (excluding spines), tapering to acute tip.

**Antennal peduncles** (Figure 1a) overreaching distal corneal margin by about 0.8–0.9 length of ultimate segment, overreaching distal margin of antennal peduncle; third segment unarmed, slightly broadened distally, about 4 times longer than distal width, subequal in length to penultimate segment, with a single setae dorsomedially and subdistal tuft of paired setae; second segment unarmed, with tuft of short setae on dorsodistal margin and second tuft near proximal margin; basal segment unarmed except for dentate ventrodistal outer border, with distal tuft of setae.

**Antennal peduncles** (Figure 1a,c) overreaching distal corneal margin by 0.3–0.4 length of fifth segment; fifth segment with tufts of long stiff setae on dorsal and ventral outer margins, unarmed; fourth segment with disto‐outer spine and associate tuft of long setae; tuft of setae on distomesial margin; third segment unarmed; second segment with distolateral outer process stout and acute; smaller but strong spine on distomesial angle; mesial face with 3–4 conspicuous distal spines, with dense short plumose setae associated with the spines; first segment dentate on outer and inner anterior margins; plumose short setae present on lateral and ventral surfaces. **Antennal acicle** triangular, almost reaching distal margin of fourth peduncular segment, bearing simple strong terminal spine plus usually 5–6 spines along mesial margin, and tufts of simple setae on both mesial and lateral margins. **Antennal flagellum** short and robust, twice length of shield, reaching base (male) or distal part (female) of dactylus of left cheliped, consisting of 28–32 articles with paired long ventrolateral setae and shorter setae on dorsal and ventral surfaces.

**Third maxilliped** (Figure 1d) basis with 1–2 small spines; ischium with *crista dentata* bearing 2 prominent spines at distal half and 2 smaller spines proximally; merus longer than carpus; ischium and merus with rows of long setae on lateral margins; carpus, propodus and dactylus with dense tufts of thick, very long setae in dorsodistal and dorso‐median margin, concealing part of segments; exopod peduncle reaching 1/3 of endopod carpal length, with row of long setae on mesial margin.

**Male left cheliped** (Figures 2a,b,d,e, 3d,e, 4d) much larger than right (Figure 1e). **Dactylus** about 1.4 times longer than palm measured along upper margin, proximally broadened, slightly arched, ending in large calcareous claw, crossing tip of fixed finger; upper inner and outer margins well defined by row of strong spines; one incomplete row of smaller subacute tubercles running parallel to upper margin, covering proximal 3/4 of dactylus length; outer surface flattened, covered with small evenly distributed obtuse tubercles reaching lower margin; cutting edge sinuous, with row of calcareous teeth of various size, biggest in proximal area; lower margin with tufts of stiff setae; rest of outer surface with some sparse setae associated with tubercles; inner surface smooth and glabrous, except for row of rounded tubercles in medial zone running parallel to upper margin, defining a concave area between them. **Fixed finger** equilateral triangle shaped, not delimited by a concavity proximo‐ventrally; outer surface covered with evenly distributed small acute tubercles increasing in size distally; cutting edge sinuous, with single row of various sized teeth, biggest medially, and row of spaced tufts of stiff setae below it; proximal area wide, not depressed; lower margin straight, defined buy rows of low rounded tubercles; inner surface without setae and almost smooth. **Palm** robust, about 1.1 times higher than long (maximum height—max. medial length); upper margin slightly convex, shorter than carpus, defined by row of well‐developed spines of similar size with associate short setae; upper inner margin defined by row of smaller but strong spines; space between rows slightly widening distally; outer surface medially inflated; upper outer part (below row of spines on upper margin) slightly concave proximally; rest of palm outer surface with small sharp spines, larger in upper half, larger ones arranged in diffuse longitudinal rows; distinct row of well‐developed spines starting in proximal lower margin, running obliquely to proximal margin, continuing then with central longitudinal diffuse row of smaller spines decreasing in size distally; lower margin almost straight in outer view, defined by rows of well‐developed spines increasing in size proximally, where join proximal row; proximo‐ventral area with greater accumulation of spines on slightly protruding flat area; lower margin keeled and sinusoidal in ventral view (Figure 3d‐f); inner surface glabrous, slightly inflated medially, concave proximally, covered with poorly developed rounded tubercles, of similar sizes, becoming acute tubercles near upper margin; well‐defined smooth concave area adjacent to inner lower margin. **Carpus** as long as high (Figure 2a,b), 1.3 times longer than palm upper margin; upper outer margin defined by row of strong spines increasing in size distally, with row of smaller spines on upper inner margin; outer surface convex, covered with small tubercles becoming spines medially, with sparse short setae; broad shallow concave area present just below upper outer margin, becoming deeper proximally; lower margin defined by row of small spines, biggest subdistal; disto‐outer anterior border bearing series of small spines submarginally; inner surface covered with sparse low acute tubercles becoming spines mesially, almost glabrous; distal margin dentate, with row of short setae. **Merus** 1.4 times longer than high (Figure 2a,b,e,g), subtriangular in dorsal view; distal margin with sparse small spines, largest on ventrolateral area, with row of short simple setae; dorsal surface with row of obtuse spines, increasing in size distally, accompanied with long plumose setae; lateral surface almost smooth except for small spines adjacent to dorsolateral and ventrolateral margins, glabrous; spinose transverse furrow subdistally, with associated short setae; ventrolateral margin denticulate with strong spines, biggest medially, and tufts of plumose setae; proximal half markedly concave, defining a wide depression at ventral area (Figures 2g, 4d); mesial face with weakly calcified u‐shaped patch, distally divided by deep transverse furrow dorsally bearing small denticulate protuberances (Figure 2e) and tufts of long thick setae; distal mesial part divided into dorsal, central and ventral lobes by median clefts; dorsal lobe with distal margin bearing spines of similar size and tufts of medium‐size setae; central lobe small, with distal margin smooth; ventral lobe with ventral margin defined by rows of strong spines, and tufts of long plumose setae. **Ischium** with transverse row of small spines on distolateral margin and row of acute tubercles on ventral margin (Figure 2e).

**Female left cheliped** (Figures 2c, 3e,f) differs from male in the following features: **Palm** oval, globose, 1.2 times longer than high; outer surface with more developed tubercles and spines, but arranged in similar way, with well‐defined longitudinal rows of spines at middle area and at upper half; lower margin convex throughout, with marginal flat area extending from proximal margin until beginning of fixed finger. **Carpus** with strong spines on distal upper and lower margin, and longitudinal row of spines medially. **Merus** with spines of dorsal margin larger than in males; median cleft almost inconspicuous.

**Right cheliped** (Figure 1e) appreciably shorter than left, robust, reaching proximal margin of palm of left; dactylus and fixed finger with narrow hiatus, both terminating in small calcareous claws. **Dactylus** slightly more than 2.0 times longer than palm (measured along mesial margin), gently arched; upper inner and outer margins defined by rows of spines with associated long abundant setae; outer surface slightly convex, covered with irregular rows of spines in upper half; lower half almost smooth; cutting edge with row of small calcareous teeth, terminating in small calcareous claw and tufts of setae parallel to cutting edge; inner surface smooth except for two rows of tufts of setae parallel to upper margin and cutting edge. **Fixed finger** not broadened proximally, with rows of well‐developed spines; inner surface smooth, with tufts of simple setae on palm and two rows of stout setae on fixed finger. **Palm** upper outer margin defined by a row of spines; outer surface convex, with rows of small spines, obscured by tufts of long setae; lower margin defined by small obtuse subacute tubercles. **Carpus** widened distally, with row of strong spines on upper outer margin increasing in size distally; outer surface with row of rounded tubercles medially, and second row near lower margin; space between upper spinose margin and medial tuberculate row, smooth and markedly concave; lower margin smooth, with sparse tufts of simple setae; inner surface with distal margin dentate and associated setae, rest of inner surface smooth, and almost glabrous. **Merus** distal margin with spines of different size, largest on dorsal area, with long simple setae; dorsal margin weakly delimited by a row of small subacute tubercles becoming obtuse small spines distally and tufts of long plumose setae; lateral surface with small spines adjacent to ventrolateral margin; transverse furrow subdistally with sparse long setae, rest of lateral surface smooth, with sparse setae; ventrolateral margin delimited by a row of small obtuse spines increasing in size distally, and tufts of long plumose setae; mesial face with weakly calcified patch proximally, smooth, with ventromesial margin defined by row of small spines decreasing in size distally. **Ischium** with short transverse row of small spines on distolateral surface and longitudinal row of acute tubercles on ventromesial margin, with tufts of setae associated with spines and tubercles.

**Second and third pereopods** (Figure 1f) moderately stout, subequal in length, overreaching distal margin of left cheliped when fully extended. **Dactylus** about 1.2 times longer than propodus, weakly curved, terminating in moderately small corneous claw; upper and lower margins unarmed, with rows of long simple setae more numerous in upper outer margins; outer surface without longitudinal sulcus medially, with row of sparse long simple setae medially; inner surface with longitudinal rows of long stout setae adjacent to upper and lower margins, with additional proximal tuft. **Propodus** about same length as merus (second) or 1.2 times longer (third), with upper margin defined by row of spinules (second) or tiny blunt tubercles (third), with row of long setae; lateral surfaces each with longitudinal row of setae arising from tiny low protuberances below upper margin and second inconspicuous row below midline; lower margins smooth with scarce short setae. **Carpus** upper margin with row of strong spines of similar size and rows of plumose setae dorsally (second), or with small spines on upper margin, increasing in size distally (third); lateral surfaces with low tubercles arranged in two rows, and tufts of setae associated with them; ventral surfaces and distolateral margin with tufts of long setae. **Merus** upper margin faintly dentate (second), almost smooth (third); lower margin defined by row of small spines, with small distal spine (second), or almost smooth, without distal spine (third); tufts of long plumose setae on upper and lower surfaces. **Ischium** unarmed, with long setae on distal margin.

**Fourth pereiopods** (Figure 2f). **Dactylus** with row of 10 minute spiniform setae on distal part of ventral margin. **Propodus** suboval, with distodorsal spine and numerous setae on dorsal margin; propodal rasp consisting of 5–6 rows of corneous scales, covering distoventral part including fixed finger. **Carpus** with distodorsal spine; rest of segments unarmed, with clumps of long plumose setae.

**Fifth pereiopods. Propodus** 0.7 times shorter than merus and 1.2 times longer than carpus; group of subacute corneous scales in distodorsal surface of propodus and smaller ones in dactylus and fixed finger; very long clumps of strong simple setae.

Male unpaired left **pleopods** 2–5 present, uniramous, marginally setose. Female with paired **gonopores**, unpaired 2–4 pleopods well developed, birramous; fifth pleopods without exopod, as in male.

**Telson** (Figure 1g) with median cleft usually developed but not pronounced, markedly asymmetrical; left posterior lobe with two strong terminal spines and with row of spines of different sizes on lateral margin, becoming blunt anteriorly; oblique terminal margin with few smaller spines; additional row of larger spines along the ventral surface of the lateral margin; right posterior lobe with two terminal medium‐size spines and row of small spines on less oblique terminal margin; spines on lateral margin decreasing in size anteriorly, extending onto posterior half of lateral margin.

**Coloration:** Green corneas spotted with yellow; ocular peduncles pale orange with brownish orange rings below corneas and triangular stain of the same color projecting from base toward apex as narrow line. Antennules and antennae background pale orange with brownish orange spots; shield with orange background and brownish orange spots; ocular acicles and intercalary rostriform process also brownish orange. Left cheliped uniformly orange colored in background, with spines and tubercles tinged with brownish red, giving characteristic reddish‐orange appearance. Right cheliped similar to left, with whitish areas at distal part of dactylus and fixed finger, proximal part of palm and area of carpus between upper and medial row of spines. Pereiopods with pale orange background; merus with one distal and a second subdistal vermillion red rings; carpus with vermillion red stains at proximal and distal upper margins, and ring of the same color subproximally; propodus with vermillion red ring medially; dactylus with faint brownish orange proximal area.

**Etymology:** The specific name “*erythromanus*” refer to the coloration of the fresh specimens, especially the left chelae, with a uniform reddish‐orange color.

**Habitat**: Sandy bottom along shallow subtidal areas, from 15 m up to 31 m. There is still little information about the bathymetric range of this species, but it seems to be more abundant around 15 m.

**Distribution:** Known so far from two Iberian localities, Baños del Carmen and Cabo Pino, both located in the southeastern coasts of the Iberian Peninsula (Málaga) and one, Kenitra, in the northern Moroccan coasts.

**Remarks:** Compared to the current known local species in the Iberian Peninsula, and therefore in the whole of the European Atlantic waters, *Diogenes erythromanus* sp. nov. is readily identifiable by the shape of the left cheliped of both sexes, with an almost straight lower margin (outer view) in males and a globose circumference in females. Moreover, the presence of a small spine at disto‐outer margin of antennal segment 4 is only known to occur in *D. erythromanus* sp. nov. and *D. arguinensis* sp. nov. (described below). Only the later species shares some characters with *Diogenes erythromanus* sp. nov., although the shape of the left cheliped shows some valuable differences. The dactylus is noticeably longer in *D. arguinensis* sp. nov., flattened and in adult individuals, markedly twisted in dorsal view, while in *D. erythromanus* sp. nov. there is no torsion of the dactylus and is only slightly flattened. Moreover, the palm is higher than long in *D. erythromanus* sp. nov, while in *D. arguinensis* sp. nov. is always longer than high and with the lower margin usually markedly concave.

**FIGURE 5:**
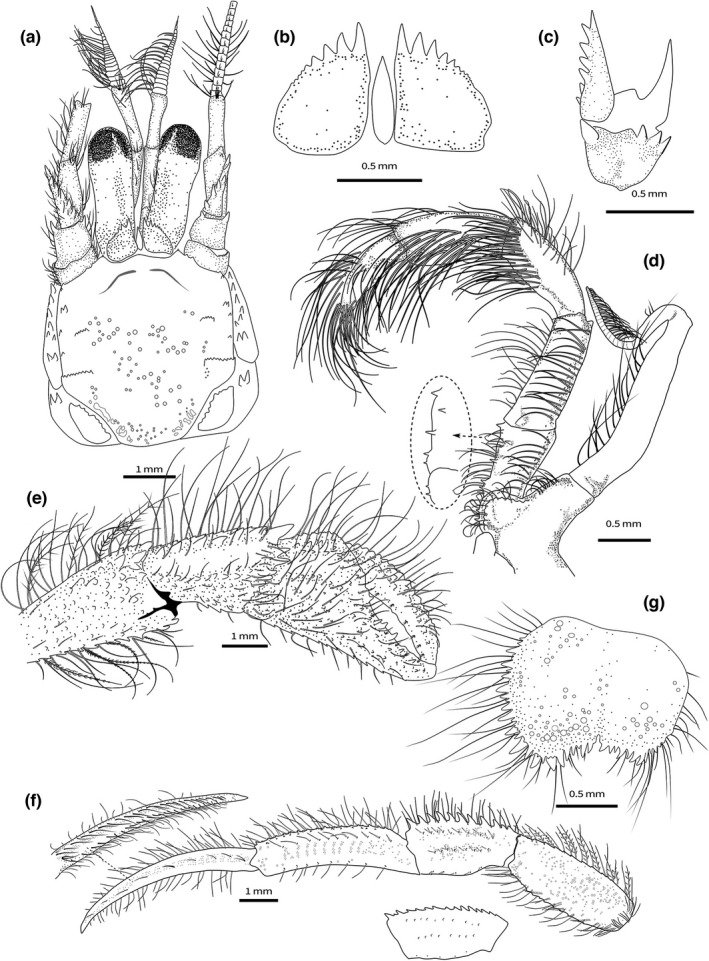
*Diogenes arguinensis* sp. nov. **Morocco**, ♂ 2.6 mm, paratype, (IEO‐CD‐CCLME12/2577‐1): (a) Anterior part of carapace and cephalic appendages, dorsal view; (b) ocular acicles and intercalary rostriform process, dorsal view; (c) right antenna, mesial face; (d) left maxilliped 3, (inset: detail of the spines, ventral view); (e) right cheliped, dorsal view; (f) left pereiopod 2 (insets‐ variations in P3 carpus; dactylus inner view); (g) telson, dorsal view. (Scales: a, e‐f = 1 mm; b‐d, g = 0.5 mm)

**FIGURE 6:**
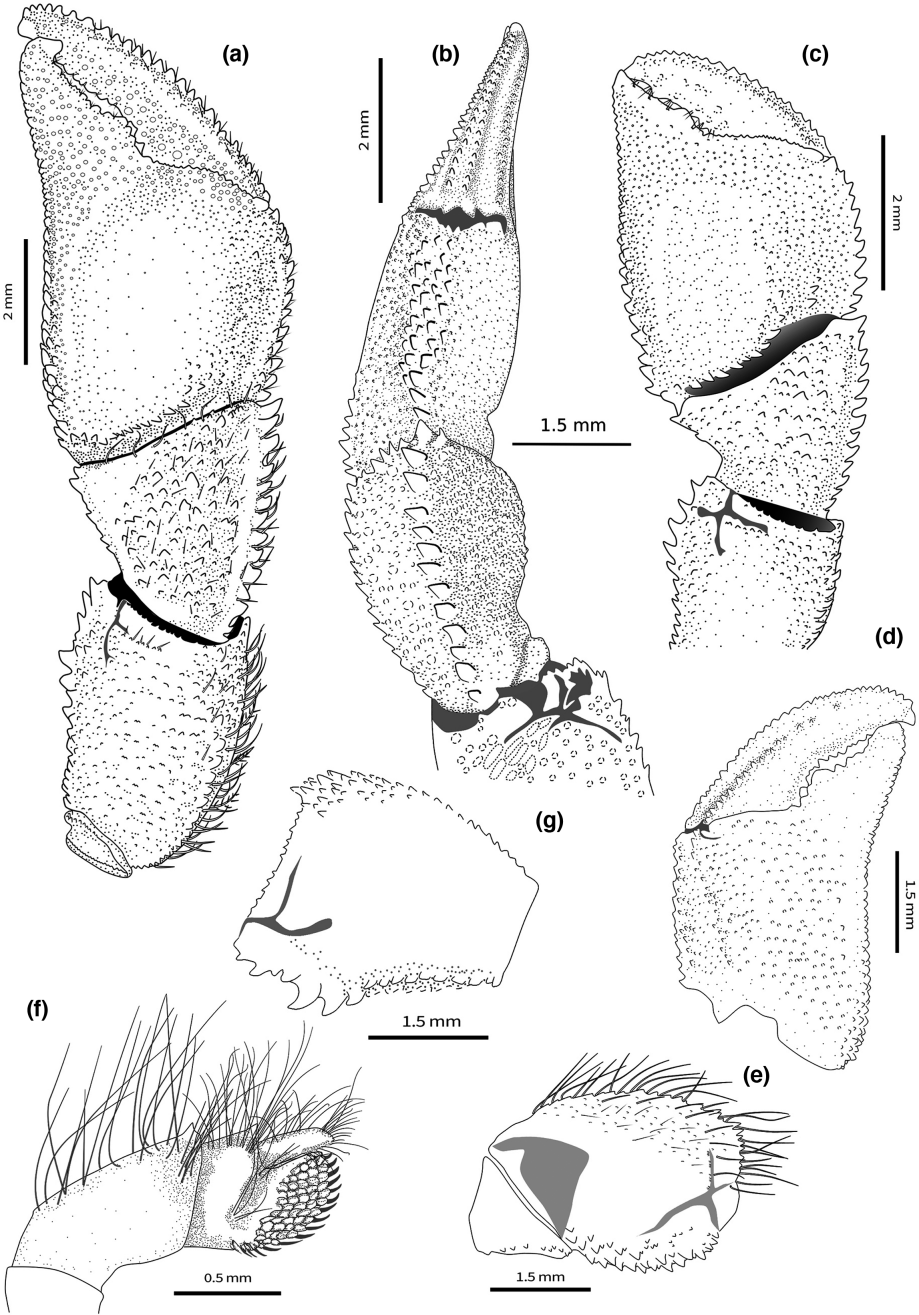
*Diogenes arguinensis* sp. nov. **Mauritania**, ♂ 3.7 mm, holotype, (IEO‐CD‐CCLME12/2572): (a) Male left cheliped, outer view; (b) left cheliped, dorsal view; (d) left cheliped propodus inner surface; (e) left cheliped merus, mesial view; (f) left pereiopod 4, outer view; *Diogenes arguinensis* sp. nov. **Spain**, ♀ 3.1 mm, (IEO‐CD‐BR‐2925); (c) left cheliped, outer view, (g) merus, outer view. (Scales: a–c = 2 mm; d, e, g = 1.5 mm; f = 0.5 mm)

*Diogenes arguinensis* sp. nov.

(Figures 3g‐i, 4e‐k, 5a‐g, 6a‐g).

LSID:zoobank.org:act:D82CB52C‐E8C7‐4613‐9C28‐85CDB5E70624.

**Holotype:** ♂ 3.7 mm, (IEO‐CD‐CCLME12/2572), **Mauritania**: off Cape Timiris, 19°19'18.5"N, 16°46'16.0"W, sand, 34 m, May 24, 2012.

**Paratypes:** 1♂ (IEO‐CD‐CCLME12/2575), **Mauritania:** off Cap Timiris, 19°19'18.5"N, 16°46'16.0"W, sand, 30 m, June 08 2012; 1♂ (IEO‐CD‐CCLME12 2577‐1), **Morocco:** off Chbika, 28°19'26.0"N, 11°40'23.2"W, sand, subtidal, 40 m, July 1, 2012; 1♀, (IEO‐CD‐CCLME12 2576‐1) near Tarfaya, 28°01'51.2"N, 12°41'34.1"W, sand, subtidal, 59 m, June 29, 2012; 1♀(IEO‐CD‐BR‐2925), **Spain:** Cabo Pino, Mijas, Málaga, 36⁰29’42’’N, 4⁰39’36’’W, fine sand, subtidal, 15 m, September 23, 2009.

Other material: **Spain:** Doñana National Park, Huelva, 1♂ (IEOCD‐BR/2681), 37º07.111'N, 6º48.636’ W, sand, subtidal, 3 m, May 17, 2019; 1♂ (IEOCD‐BR/2682), 37º04.582’ N, 6º45.608’ W, sand, subtidal, 8 m, May 15, 2019; Cabo Pino, Mijas, Málaga, 6♂, 8♀ (IEO‐CD‐BR‐2918‐20; 2922‐24; 2926‐32), 36⁰29’42’’N, 4⁰39’36’’W, fine sand, subtidal, 15 m, September 23, 2009; La Carihuela, Torremolinos, Málaga,1♂, 4♀ (IEOCD‐BR/2683‐85), 36º36'28.2''N, 4º30'13.6''W, sand, subtidal, 2–4 m, December 11, 2019; **Morocco**: 4 ♂, 1♀, (IEO‐CD‐CCLME12 2576‐2) near Tarfaya, 28°01'51.2"N, 12°41'34.1"W, sand, subtidal, 59 m, June 29, 2012; 4♂ (IEO‐CD‐CCLME12 2577‐2) off Chbika, 28°19'26.0"N, 11°40'23.2"W, sand, subtidal, 40 m, July 1, 2012; 1♀ (IEO‐CD‐CCL;E11 690‐1) off Dajla, 23°25'50.5"N, 16°46'47.3"W, sand, subtidal, 31 m, November 22, 2011; **Mauritania**: 2♂, (IEO‐CD‐CCLME12/2573) South of Cape Timiris, 19°01'15.6"N, 16°23'51.7"W, sand, subtidal, 21 m, June 03, 2012.

**Description: Shield** (Figure 5a) subquadrate, longer than broad, not vaulted; rostral lobe narrowly rounded, exceeded by lateral projections, which are triangular, acutely pointed, with a single spine in the apex; anterior margins between rostral lobe and lateral projections markedly concave; anterolateral margins sloping, with 1–2 spines near lateral projections; anterolateral angles rounded, usually with 1 terminal and 1 subterminal spines; lateral margins slightly convex, with posterior halves each usually cut by one transverse spinulose ridge extending onto lateral surface of shield; posterior margin truncate; dorsal surface with additional short transverse rows of small tubercles and tufts of short stiff setae, additional longitudinal row of 2–3 spines adjacent to lateral margin in distal half. Branchiostegites with row of 6+1 paired strong spines on dorsal margin. Posterolateral plates not well calcified, unarmed.

**Ocular peduncles** (including corneas) about 0.6–0.7 times as long as shield, moderately stout, slightly narrowed medially; corneas not dilated, corneal diameter about 0.3 peduncular length; row of short plumose setae over mesial margin of peduncles. **Ocular acicles** (Figure 5a,b) roundly triangular, with anterior margins convex; anterior margin bearing 5–6 strong acute distal spines widely spaced and 4–5 small spines (sometimes reduced to small tubercles), decreasing in size toward outer margin, innermost biggest; rest of latero‐anterior outer margin smooth, occupying about 1/3 of anterior margin. **Intercalary rostriform process** simple, small but strong, slightly shorter than or subequal in length to ocular acicles (excluding spines), tapering to acute tip.

**Antennular peduncles** (Figure 5a) overreaching distal corneal margin by about 0.8 of length of ultimate segment, slightly overreaching the distal margin of fifth antennal segment; third segment unarmed, slightly broadened distally, about 4 times longer than distal width, subequal in length to penultimate segment, with a row of 2–3 simple long setae on dorsal margin and tuft of smaller setae distally; second segment unarmed, with row of short setae on dorsal margin and distal tuft of short ones; basal segment unarmed with distal tuft of short setae.

**Antennal peduncles** (Figure 5a,c) overreaching distal corneal margin by 0.7 length of fifth segment; fifth segment with row of long stiff setae on lateral and ventral outer margins, and dorsodistal tuft of short ones, unarmed; fourth segment with small spine subdistally, and row of long stiff setae on lateral and ventral outer margins, with additional tuft of long setae dorsodistally; third segment unarmed; second segment with distolateral outer process stout and acute; smaller but strong spine on distomesial angle; rows of setae on lateral and ventral margins; mesial face with 3 conspicuous spines on ventrodistal margin; first segment with distolateral border dentate and rows of plumose short setae on outer and inner margins. **Antennal acicle** triangular, reaching the distal margin of fourth peduncular segment, bearing a simple strong terminal spine plus usually 5–6 spines along mesial margin, and tufts of simple setae on both mesial and lateral margins. **Antennal flagellum** short and robust, slightly more than twice length of shield, reaching the base (male) or distal part (female) of dactylus of left cheliped, consisting of 27–31 articles with paired long ventrolateral setae and shorter setae on dorsal and ventral surfaces.

**Third maxilliped** (Figure 5d) slender; basis with 2 small spines; ischium with *crista dentata* bearing 5 prominent (3 distal and 2 proximal) spines; merus longer than carpus; ischium and merus with rows of setae on ventral margin and sparse setae on lateral margin; carpus, propodus and dactylus with dense tufts of thick, very long setae on ventrodistal margin combined with patches of dense shorter ones; exopod peduncle slightly overreaching distal part of merus; inner margin with row of long setae.

**Male left cheliped** (Figure 3g,h, 4e‐k, 6a,b,d) much larger than right (Figure 5e). **Dactylus** 1.4–1.6 times longer than upper margin of palm, flattened, slightly arched in dorsal view and curved outwards distally, ending in calcareous claw, crossing tip of fixed finger; in large specimens, dactylus clearly overreaches tip of fixed finger (see variability in remarks and Figure 4f‐k); upper inner and outer margins well defined by row of large, spines; one row of small subacute tubercles running parallel to upper margin; outer surface flattened, covered of small evenly distributed rounded tubercles except in central part, that is smooth; cutting edge sinuous, with row of calcareous teeth of similar size; lower margin with tufts of short setae; inner surface with row of acute tubercles in upper half, running parallel to upper margin, defining a markedly depressed area between them. **Fixed finger** isosceles triangle shaped, flattened, delimited by deep concavity extending toward proximal area running near lower margin; outer surface covered with evenly distributed small rounded tubercles; cutting edge sinuous, with single row of various sized teeth, biggest in proximal area, and row of spaced tufts of short setae below it; lower margin with 2–3 rows of small rounded tubercles extending onto palm; inner surface with scarce tufts of setae, almost smooth. **Palm** elongate, robust, 1.2 times longer than high (max. medial length without fixed finger—maximum high); upper margin 1.4 times shorter than carpus, defined by row of strong spines of similar size and row of short setae; upper inner margin defined by row of smaller spines; space between rows slightly widening distally; outer surface inflated at proximo‐medial part, with markedly concave area defining the inflection change at base of fixed finger, running parallel to lower margin reaching proximo‐ventral margin; rest of palm outer surface with blunt small tubercles; distinct row of spines starting in proximal lower margin, running obliquely to proximal margin, with short row of spines continuing proximo‐distally; lower margin concave to variable degree in outer view (Figure 4f‐k), defined by rows of obtuse spines decreasing in size distally; lower margin keeled and slightly sinusoidal in ventral view (Figure 3g,h); inner surface covered with poorly developed rounded tubercles, of similar sizes, with concave area below lower margin; pilosity absent. **Carpus** subtriangular, as long (upper margin) as high (distal border) (Figure 6a); distal half much wider and higher than proximal; outer surface convex, covered with rounded tubercles increasing in size toward central area, where they become spinose, almost disappearing in lower and upper margins; upper outer margin defined by single sinuous row of strong spines increasing in size distally; inner margin by row of spinose tubercles increasing in size distally; pilosity scarce, with short setae only associated with tubercles of lower, central, and upper zones; broad shallow depression present in upper outer side, just below upper margin, becoming deeper and widening proximally; lower margin with sparse small spines increasing in size distally, the biggest submarginal; disto‐outer anterior border bearing a series of small spines submarginally, and row of sparse simple setae; inner surface covered with sparse low rounded tubercles and series of plumose setae. **Merus** longer than high (Figure 6a,e,g) subtriangular in dorsal view; distal margin spinose, largest spines on dorsal margin, with sparse short setae; dorsal surface with row of obtuse spines decreasing in size proximally, accompanied with medium‐size plumose setae; lateral surface with irregular rows of spines adjacent to dorsolateral and ventrolateral margins; rest of lateral surface with spinulose protuberances arranged in transverse rows; lateral surface glabrous; shallow spinose transverse furrow subdistally, with associated very short sparse setae; ventrolateral margin dentate, with strong spines, biggest medially, and tufts of plumose setae; proximal half tuberculated and markedly concave, defining a wide depression at ventral area (Figures 4e, 6g); mesial face with weakly calcified subtriangular patch, distally divided by shallow transverse furrow dorsally bearing small spinulose protuberances (Figure 6e) and sparse long simple setae; distal mesial part divided into dorsal, central and ventral lobes by median clefts; dorsal lobe with distal margin bearing spines of similar size and tufts of long setae; central lobe small, with distal margin smooth; ventral lobe with ventral margin defined by rows of strong spines, without setae. **Ischium** with transverse row of small spines on distolateral outer margin and longitudinal row of spines on ventral mesial margin (Figure 6a, e).

F**emale left cheliped** (Figures 3i, 6c) differs from male in the following features: **Palm** globose, 1.2 times higher than long; upper and lower palmar margins gently convex. **Dactylus** shorter, similar in length to upper margin of palm and only slightly flattened. **Fixed finger** triangular, broadened proximally. **Carpus** lower margin with proximal half smooth and markedly concave, forming a wide sinus. **Merus** dorsal margin with smaller spines than in males, similar in size to other spines in dorsal margin.

**Right cheliped** (Figure 5e) appreciably shorter than left, reaching proximal margin of palm of left; dactylus and fixed finger with relatively narrow hiatus, both terminating in small calcareous claws. **Dactylus** slightly more than 2.0 times longer than palm (measured along mesial margin), gently arched; upper inner and outer margins defined by rows of small spines and rows of long setae; outer surface slightly convex, with row of spines below spinose row at upper margin; cutting edge with row of small calcareous teeth, regular in size, and tufts of short setae parallel to cutting edge; inner surface smooth except for two rows of tufts of stout setae on medial and lower areas. **Fixed finger** not markedly broadened proximally; cutting edge with row of small, subacute calcareous teeth and row of tufts of long stout setae parallel to cutting edge; rest of outer surface of fixed finger with several rows of spines, bigger near masticatory border; inner surface smooth with two rows of tufts of setae on medial and lower margins, slightly continuing into the palm inner surface. **Palm** upper outer margin defined by a row of obtuse spines; outer surface slightly convex, with irregular rows of small spines and associated long setae; lower margin defined by small obtuse spines. **Carpus** widened distally; disto‐outer margin spinose, with spines increasing in size dorsally; upper outer margin with row of strong spines increasing in size distally; second sinuous row of spines medially, delimiting a broad smooth depression with upper row; rest of outer surface with small spines and sparse long simple setae; lower surface not well defined, with some sparse long simple setae; inner surface with distal margin dentate, rest of inner surface smooth and glabrous. **Merus** distal margin unarmed and glabrous; dorsal margin delimited by row of spines increasing in size distally and tufts of long plumose setae; lateral surface with small spines increasing in size distally, adjacent to dorsal and ventral rows, glabrous except for rows of short setae below dorsal area; transverse furrow absent; rest of lateral surface with low tubercles and sparse short setae; ventrolateral margin delimited by row of spines increasing in size distally, and tufts of long plumose setae; mesial face with weakly calcified patch proximally, smooth except for some low tubercles adjacent to upper margin and small spines near ventral margin, glabrous; ventromesial margin defined by row of small spines increasing in size proximally. **Ischium** with tuberculate proximal outer margin and with small spines on ventrodistal margin; ventromesial margin with distal spine and tufts of setae on ventral surface.

**Second and third pereopods** (Figure 5f) moderately stout, subequal in length. **Dactylus** about 1.2 times as long as propodus, weakly curved and slightly twisted distally; terminating in moderately small corneous claw; upper and lower surfaces unarmed, with rows of long simple setae more numerous at upper surface; outer surface with shallow longitudinal medial sulcus, bearing sparse simple setae; inner surface with longitudinal rows of long stout setae adjacent to upper and lower margins, and medial tuft of setae proximally. **Propodus** slightly longer than merus (second) or markedly longer (third), with upper margin defined by row of small spines (second) or spinules (third), and with row of long setae; lateral surfaces each with longitudinal row of setae arising from tiny low protuberances near upper margin and second inconspicuous row near lower margin; lower margins smooth. **Carpus** upper margin with row of large spines of similar size, and with row of sparse plumose setae dorsally (second), or with smaller spines on upper margin with sparse short setae (third); lateral surfaces smooth, with two rows of small tubercles with associated tufts of short setae; ventral surfaces smooth, with sparse short setae. **Merus** upper margin with small obtuse tubercles (second), or tiny tubercles (third); lower margin with sparse small spines and small distal spine (second), or almost smooth, without distal spine (third); tufts of long plumose setae on upper and lower surfaces. **Ischium** unarmed, with long setae on upper and lower margins.

**Fourth pereiopods** (Figure 6f). **Dactylus** with row of 11–12 minute spiniform setae on distal part of ventral margin. **Propodus** oval, with numerous setae on unarmed dorsal margin; propodal rasp consisting of 5–6 rows of corneous scales, covering distoventral part including fixed finger. **Carpus** with distodorsal spine, with clumps of long plumose setae. **Merus** unarmed.

**Fifth pereiopods. Propodus** 1.4 times shorter than merus and 1.3 times longer than carpus; group of subacute corneous scales in distodorsal surface of propodus and smaller ones in dactylus and fixed finger; very long clumps of strong simple setae.

Male unpaired left **pleopods** 2–5 present, uniramous, marginally setose. Female with paired **gonopores**, unpaired 2–4 pleopods well developed, biramous; fifth pleopods without exopod, as in male.

**Telson** (Figure 5g) slightly asymmetrical, median cleft small and shallow; left posterior lobe slightly larger than right, with strong terminal spines and with row of slightly smaller spines on lateral margin decreasing in size anteriorly; oblique terminal margin with smaller spines; few additional larger spines along the ventral surface of lateral margin; right posterior lobe with row of small spines on less oblique terminal margin, becoming blunt tubercles on posterior half of lateral margin.

**Coloration:** Unknown. All available specimens were preserved in alcohol.

**Etymology:** The name of the species pays tribute to the area of origin of the specimen on which its description is based, off the Cape Timiris, near the National Park Banc D'Arguin, a protected area of singular value on the Mauritanian coast, where the first author spent several years and of which he keeps a pleasant memory.

**Habitat**: Sandy beaches along shallow subtidal areas, up to 60 m.

**Distribution:** The species has been recollected so far from several Atlanto‐Mediterranean localities in the Iberian Peninsula (Málaga: Mijas and Torremolinos; Huelva: Doñana National Park), and from northwestern African coasts (Morocco and Mauritania).

**Remarks:** As it happens in other *Diogenes* species, the shape and size of the left cheliped seems to be highly variable in this species. The main variations observed in the samples analyzed have been summarized in Figure 4(f–k). Besides the possible variations, the combination of the flattened and curved dactylus and fixed finger in the shape of an isosceles triangle, the outer surface of palm with a concavity defining the inflection change along the base of the fixed finger and running then parallel to the lower margin, the lower margin of palm concave at distal half, and the carpus lower margin without proximal sinus, can be considered persistent for males in all samples, and therefore, representative of the species. Female individuals have shown less variability, with only some differences in the development of spines and tubercles, generally, size related. We acknowledge, as one of the reviewers of this paper also proposed, that the new species shares some similarities with the *Diogenes pugilator* var. *cristata* (Balss, 1921) as described by Rossignol (1962), especially at cheliped level. Despite the undoubted similarities found between the chelipeds, the drawings of the cephalothorax included in his work shows notable differences. Attempts to locate the specimens used by Rossignol in his descriptions have been unsuccessful. In addition, it is difficult to form an opinion on the varieties mentioned or described by Balss (1921) because of the brevity of his diagnoses and the absence of figures. Forest (1955) mentioned that unfortunately the types of the new Balss varieties were destroyed during the war, and that the *cristata* variety is possibly related to *D*. *denticulatus* (and the *subcristata* variety to a separate species). Therefore, we do not have enough evidence to decide whether or not it could be the same species, which is why the resurrection of the Rossignol variety has been ruled out, describing this form as a new species. On the other hand, according to Rossignol (1957, 1962), this variety can be easily obtained by diving at shallow depths along the beach of Pointe‐Noire, particularly near the mouth of the Songolo River. Our specimens are frequent between 15 and 25 m (rare at 5 m) in the area of Cabo Pino (Málaga, Spain).

## 4 DISCUSSION

### 4.1 Taxonomic remarks

*Diogenes erythromanus* sp. nov. and *D. arguinensis* sp. nov. are referred to the “edwardsii” group, defined by the presence of the simple intercalary rostriform process, the antennal peduncle distinctly longer than the ocular peduncle, and the presence of paired long setae inserted on the ventral surfaces of articles of the antennal flagellum (Asakura & Tachinawa, 2010). Ten other species of *Diogenes* occur in the East Atlantic Ocean, all of them showing enough diagnostic characters to distinguish them from the species described here.

The combination of short and reduced intercalary rostriform process, long ocular peduncles, and the shield bearing strong spines on latero‐dorsal surfaces, is characteristic of *D. mercatoris* Forest, 1952, with no other species showing that combination. The intercalary rostriform process is well developed but spinose in *D. denticulatus* Chevreux & Bouvier, 1981, and *D. ortholepis* Forest, 1961, while in the species described above, is always smooth. The evident depression on the upper face of the chelar carpus of *D. ovatus* Miers, 1881, allows to identify this species, since no other Atlantic *Diogenes* shows similar sculpture in carpus. The serration on the branchiostegite is also different in *D*. *brevirostris* Stimpson, 1858, and *D. extricatus* Stebbing, 1910, with only 2–3 spines on the posterior branchiostegite, while in *D. erythromanus* sp. nov. and *D. arguinensis* sp. nov. they are continuously serrated. The left cheliped of *D. costatus* Henderson, 1893, has an obliquely longitudinal ridge on the outer surface and the carpi of the pereiopods bear some scarce spines, never being continuously serrated. Confusion is furthermore unlikely, as *D. brevirostris*, *D. extricatus*, and *D*. *costatus* have been exclusively recorded from Atlantic South African waters.

Within the so‐called “*Diogenes pugilator* species complex,” three other species have been described previously in the same area, although all of them can be easily differentiated from the new ones by the presence in the later of a disto‐outer spine in antennal peduncle segment four, which is not present in *D. pugilator*, *D. armatus*, or *D. curvimanus*. Additionally, the ventrolateral outer margin of merus of left cheliped in *D. erythromanus* sp. nov. and *D. arguinensis* sp. nov. is markedly concave in proximal half and defined by strong spines, while in *D. curvimanus*, *D. armatus*, and *D. pugilator*, this concavity is present but less pronounced, and delimited by tubercles, nor spines (Figure 4a–e). The male specimens of *D. curvimanus* has also a unique cheliped, which is slender and long, almost without pilosity and with scarcely developed spines, showing in most of the cases only different sizes of rounded tubercles associated with the upper margin of the cheliped. The ocular acicles are continuously serrated in *D*. *armatus*, and the male left cheliped outer surface bears strong spines, with abundant pilosity and long setae, while in the species described above the ocular acicles are only partially serrated, the male left cheliped has the outer surface less spinose and the pilosity is sparse and short. The palm of male left cheliped of *D. pugilator* is globose, with the outer surface markedly inflated and covered by small spinose tubercles, while in the species described here, the palm is not inflated and has spines of different sizes in outer surface. Moreover, the lower inner margin of palm of left cheliped shows clear differences, allowing to separate the new species from *D*. *pugilator*, *D. armatus*, and *D. curvimanus* (Figure 3a–i), even when they are inside the shell. In the new species, the lower margin is keeled and sinusoidal, in ventral view, defined by row of spines, while the other species has several rows of tubercles, defining a more or less extensive flattened tuberculated area, being in *D. pugilator* also delimited by a sinuous crest‐like row of large rounded tubercles. The new species are morphologically very close to each other, being the main diagnostic characters: The general shape of the palm of the left cheliped is slightly higher than long in *D. erythromanus* sp. nov., while in *D. arguinensis* sp. nov. is longer than high; the presence in males of *D. erythromanus* sp. nov. of a flat area with accumulation of spines on proximo‐ventral area of palm, slightly protruding ventrally, is not present in *D. arguinensis* sp. nov. (this character is even more evident in females, where the flat area extends along most of the lower margin overreaching the base of the fixed finger); the fingers of the left cheliped in *D. arguinensis* sp. nov. are markedly twisted in dorsal view, the palm outer surface is less tuberculated, and the medial row of spines is shorter than in *D. erythromanus* sp. nov.; the lower margin of palm of male cheliped is almost straight in *D. erythromanus* sp. nov., bearing well‐developed spines, while in *D. arguinensis* sp. nov. there is always a concave area at lower margin, more or less developed but always evident, bearing also markedly obtuse spines.

Differentiation of females can be more challenging, with palm of left cheliped in both species being globose with similar shape. However, the presence of a flat area along the lower margin is persistent in all specimens, making the palm of *D. erythromanus* sp. nov. slightly less rounded in shape. Moreover, the outer surface is more granulose, with larger tubercles and spines, while in *D. arguinensis* sp. nov. the appearance is smoother, although it does present tubercles, but these are low and rounded.

### 4.2 Phylogenetic analyses

**FIGURE 7:**
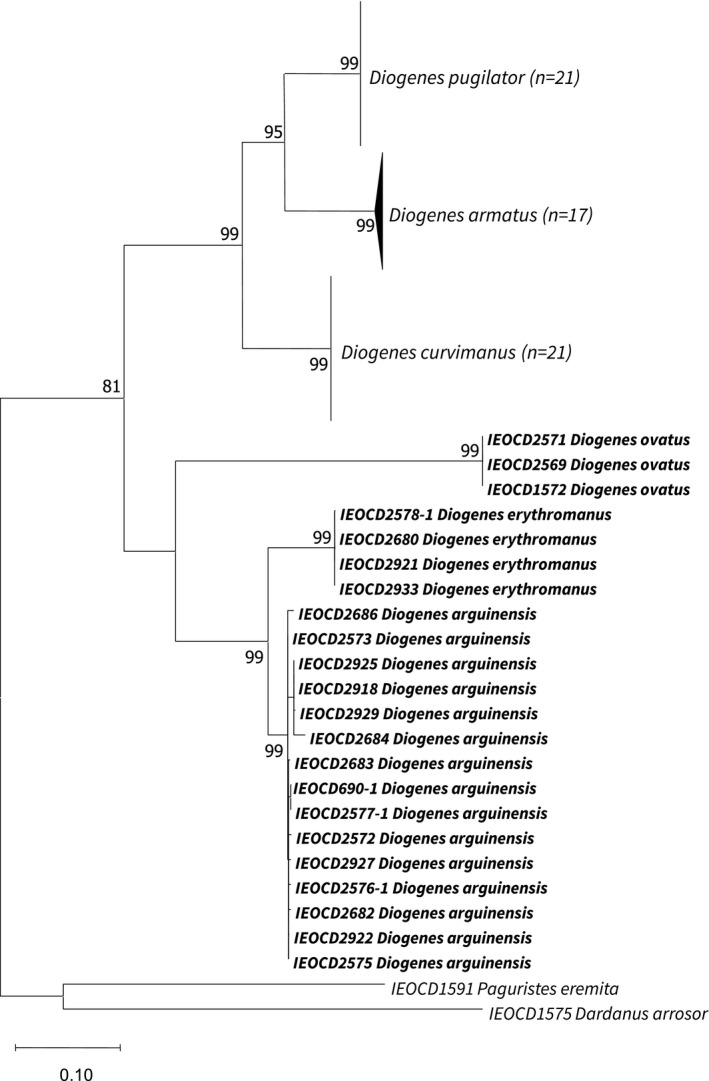
Maximum likelihood phylogenetic tree based on the concatenated mitochondrial data set (16S+COI) including available information of representatives of the genus *Diogenes*, using 1000 nonparametric bootstrap replicates. Numbers on the branches represent ML bootstrap values; only bootstrap values >70 (ML) are included. The species *Dardanus arrosor* and *Paguristes eremita* are included as outgroups

We analyzed the phylogenetic relationships among the species of *Diogenes* to test whether the new species constitute genetically separate units. The concatenated dataset includes 6 of the 10 species distributed in the eastern Atlantic, since molecular information for the other species is still limited and have been included in the individual genes datasets when available. Maximum likelihood analyses of the combined (Figure 7) and individual genes datasets (Appendix 1, Figures [Link], [Link], [Link]) all yielded similar results, recovering the two putative species as monophyletic units, with relatively long branches and strong nodal supports (Figure 7).

Results from individual gene analyses allowed to obtain a general overview of the taxonomic status of the species complex, based on the comparison with sequences obtained from NCBI/BOLD databases (Appendix 1, Figures S1, S2 and S3). For the 16S gene, only 6 sequences were available and none of them correspond to species within the study area, while for nuclear 28S gene, no sequence was available for species of this genus. However, the higher number and representatives of species among the partial sequences of the COI gene allowed to obtain an overview of the intrageneric relationships, where the new species are clustered together with *D. costatus and D. albimanus*, both species recorded from South African Atlantic waters. The analysis shows another three species grouped relatively close to the previous ones, including the Indo‐West Pacific *D. merguiensis* de Man, 1888 [in de Man, 1887–1888] and *D*. *viridis* Haig & Ball, 1988, along with *D. canaliculatus* Komai, Reshmi & AB Kumar, 2013, a species with distribution in the Indian ocean and the Red Sea. Genetic intraspecific divergence values were of 0.01 in *D. erythromanus* sp. nov., ranging between 0.00 and 0.02 within sequences belonging to *D. arguinensis* sp. nov., with divergence values between the two new species ranging from 0.07 to 0.09. Interspecific distances between the new taxa and other congeneric species ranged from 0.04 to 0.07 for *D. costatus*, 0.12 to 0.16 for *D. albimanus*, and from 0.17 to 0.22 for *D. pugilator*, *D. armatus*, and *D. curvimanus*.

There are still many gaps regarding molecular information about species of the genus with distributions that could help to explain the intrageneric relations, as well as to better understand the possible migratory displacements carried out from native areas. However, the results from the molecular phylogenies suggest a closest relationship of *D. erythromanus* sp. nov. and *D. arguinensis* sp. nov. with species of tropical affinity, rather than those of European temperate waters. Therefore, the DNA evidence agrees with separations based on morphological characters and confirm the taxonomic delimitation of the species.

### 4.3 Biogeographical implications

The presence of *D. erythromanus* sp. nov. and *D. arguinensis* sp. nov. on both sides of the Strait of Gibraltar, as well as the relationships inferred from the phylogenetic analysis, suggest a closest connection with other African congeneric species, rather than with other European ones, opening the possibility to different explanations for their presence in the Iberian Peninsula.

The first hypothesis involves the migration of the species from Africa to Europe, a process known as tropicalization (Cuesta et al., 2016). It is well known that anthropogenic activities and climate change are among the most important factors that may enhance the establishment of introduced species, as well as the poleward shift in distribution of numerous species over decades (González‐Ortegón et al., 2020), even overcoming important geographic barriers under favorable conditions (Patterson et al., 2022). The Strait of Gibraltar area has an important role as a pathway of introductions both from the Mediterranean Sea and by West African biota moving northwards into European waters, the later labeled as “African Creep” by Canning‐Clode and Carlton (2017). The number of decapod species that have followed this path in the last decades is significant (e.g., *Acantharcus posteli* (Forest, 1963), *Brachynotus atlanticus* Forest, 1957, *Callinectes pallidus* (de Rochebrune, 1883), *Cryptosoma cristatum* Brullé, 1837 [in Brullé, 1837–1839], *Lysmata uncicornis* Holthuis & Maurin, 1952, *Ogyrides rarispina* Holthuis, 1951, *Panopeus africanus* A. Milne‐Edwards, 1867, and *Xaiva mcleayi* (Barnard, 1947)), and is expected that continues to increase in the future (Encarnação et al., 2019; García‐Raso, 1985, 1993; García‐Raso & Manjón‐Cabeza, 1996; González‐Ortegón, García‐Raso, et al., 2020; Holthuis, 1977; Pozuelo et al., 1976).

Although *D. arguinensis* sp. nov. can be found in high numbers in some areas of the southeastern Iberian Peninsula, which may suggest that the species was already established in the area, the hypothesis of a recent arrival cannot be discarded. The spread of a species well adapted to its new environment can be fast, as it has happened with other species as the African Hermit crab *Pagurus mbizi* (Forest, 1955) or the African Pea crab *Afropinnotheres monodi* Manning, 1993, among others (García‐Raso et al., 2014; Perez‐Miguel et al., 2019). Based on the available data, *D. arguinensis* sp. nov. may have been present in the area at least since 2009, which would imply a development of large populations in only 13 years, which is not so much time, even if we consider the establishment and reproduction under favorable conditions.

Another hypothesis suggests a situation like that reported for the West African Fiddler crab *Afruca tangeri* (Eydoux, 1835), isolated from African populations at the time of the separation of the Iberian Peninsula from the African continent, as confirmed by the fossil records (Gibert et al., 2013). Under this scenario, *D. erythromanus* sp. nov. and *D. arguinensis* sp. nov. had always been present in the area, but for different reasons, they have gone unnoticed. The explanation would be straight forward then, agreeing with the circumstance described above, where several species have been overlooked for decades due to the consideration of *D*. *pugilator* as a highly variable species that includes all the morphotypes frequently found in European waters, part of the Mediterranean Sea and northwest Africa.

There is no clear evidence about which of them may be correct, but according with our samples, it is evident that the southern coast of Spain seems to represent the northern limit of the current distribution for both species.

### 4.4 Updated key to the presently known Atlantic species of *Diogenes* Dana, 1851

An update of the recently published “key to the presently known Atlantic species of *Diogenes* Dana, 1851” (Almón et al., 2021) to include the new species described above, is proposed.

**Table:** 

1	Intercalary rostriform process between ocular acicles reduced. Shield with oblique rows of strong spines (see Forest 1952: Figs. 1–5, Forest 1955: Fig. 14, Pl. II, 8)	***D. mercatoris*** Forest, 1952
1’	Intercalary rostriform process between ocular acicles not reduced. Spines on shield not as above	2
2	Intercalary rostriform between ocular acicles process spinose	3
2’	Intercalary rostriform process between ocular acicles smooth	4
3	Ocular peduncles not overreaching base of fifth segment of antennal peduncles. Inner border of antennal acicle concave (see Forest 1955: Fig. 13, Pl. II, fig. 7)	***D. denticulatus*** Chevreux & Bouvier, 1892
3’	Ocular peduncles long, overreaching base of fifth segment of antennal peduncle. Inner border of antennal acicle straight (see Forest 1961: Fig. 1–4)	***D. ortholepis*** Forest, 1961
4	Branchiostegites partially serrated	5
4’	Branchiostegites serrated throughout	6
5	Upper surface of carpus of left chela convex, with irregularly arranged conical tubercles; no red spot on left chela. Wide ocular acicles (see Barnard 1950: Figs 81a,c,d)	***D. brevirostris*** Stimpson, 1858^a^
5’	Upper surface of carpus of left chela flat, with two conspicuous rows of tubercles; one red spot on outer surface at propodus base of left chela. Narrow ocular acicles (see Barnard 1950: Fig. 81h)	***D. extricatus*** Stebbing, 1910^a^
6	Palm of left cheliped oval, depressed; outer surface with depression at lower region. Carpus short, with deep depression on upper face (see Forest 1955: Figs 15, 16; Pl II,9)	***D. ovatus*** Miers, 1881
6’	Palm of left cheliped not oval. Carpus without depression on upper face	7
7	Antennal peduncles segment 4 with disto‐outer spine. Propodus of eft cheliped with lower margin keeled and sinusoidal in ventral view, defined by row of spines increasing in size proximally. Merus of left cheliped with ventrolateral margin spinose, with proximal half markedly concave, defining a deep depression extending into ventral area, where pereiopods can fit in (Figs. 1A, 5A; 3D‐I; 2A, C, G, 4D, E present study)	8
7’	Antennal peduncles segment 4 unarmed. Propodus of left cheliped with lower margin with more or less extended flat tuberculated area, not keeled and sinusoidal. Ventrolateral margin of merus of left cheliped straight or weakly concave	9
8	Palm of male left cheliped higher than long, with well‐developed spines on flat area proximo‐ventrally, slightly protruding; outer surface of palm with medial longitudinal row of small spines; dactyl and fixed finger not markedly flattened; lower margin of palm almost straight defined by rows of spines. Pereiopods 2 and 3 clearly overreaching distal margin of left cheliped when fully extended (Fig. 2A, D present study)	***D. erythromanus*** sp. nov.
8’	Palm of male left cheliped longer than high, without protruding flat area proximo‐ventrally; outer surface with very short medial row of tubercles; dactyl and fixed finger markedly flattened and twisted; lower margin of palm concave, defined by markedly obtuse spines. Pereiopods 2 and 3 equal in length or slightly overreaching distal margin of left cheliped (Fig. 6A, D, present study)	***D. arguinensis*** sp. nov.
9	Palm of male left cheliped clearly longer than high, carpus frequently higher than palm; outer surface of palm finely grained or smooth. Lower margin of carpus of left cheliped long and straight distally, slightly concave proximally. Antennular peduncle shorter than antennal peduncle, ultimate segment widened distally (Forest & Guinot 1956: Fig. 3); (Almón et al., 2021 Figs. 5, 6, 7C,F)	***D*. *curvimanus*** Clément, 1874
9’	Palm of male left cheliped not clearly longer than wide, carpus about the same height as palm; outer surface of palm with tubercles or spines, not smooth. Lower margin of carpus of left cheliped convex at distal half, forming a prominent sinus proximally. Antennular peduncle subequal or longer than antennal, not markedly widened distally	10
10	Antennular and antennal peduncles subequal in length. Ocular acicles subtriangular, with 3–5 distal spines (innermost larger), rest of anterolateral outer margin with small tubercles of similar size. Outer surface of left cheliped palm medially inflated, covered with small spinose tubercles; lower inner surface of palm defined by a sinuous crest‐like row of large rounded tubercles (Almón et al., 2021 Figs. 1, 2, 7A,D)	***D*. *pugilator*** (Roux, 1829)
10’	Antennular peduncles longer than antennal peduncles. Ocular acicles with spines on whole length of anterolateral margin, or few spines on distal half, without tubercles. Outer surface of left cheliped palm not medially inflated, with at least some larger tubercles or spines defining ridges; lower inner surface of palm not defined by a sinuous crest‐like row of large rounded tubercles (Almón et al., 2021 Figs. 4A)	11
11	Ocular acicles subtriangular with 11–12 acute spines decreasing in size, innermost larger, covering entire length of anterolateral margin. Outer surface of male left cheliped palm spinose, with largest spines forming longitudinal rows. Left cheliped hirsute (Almón et al., 2021 Figs. 3, 4, 7B,E)	***D. armatus*** Almón et al., 2021
11’	Ocular acicles with few spines restricted to distal half of anterolateral margin. Outer surface of left cheliped palm almost smooth, with short, but prominent oblique granulated proximal ridge. Left cheliped glabrous (see Barnard 1950: Fig. 81e, f; Henderson 1893: Pl. 39: 7,8; Lewinsohn 1969: Fig. 6)	***D. costatus*** Henderson, 1893^a^

The form *Diogenes* sp. named by Forest in 1956 is not included here, as it has not yet been formally described as a new species. Nevertheless, the form described by Forest can be easily separated from the rest of the Atlantic species by the reduced corneas and ocular peduncles reaching behind distal margin of antennal segment 4. Forest also points out the presence of a conspicuous tooth on disto‐outer upper margin of left cheliped palm.

^a^ Species with Atlantic records restricted to South African waters only.

## 5 CONCLUSIONS

Studies devoted to the taxonomic revision of the genus *Diogenes* in the Eastern‐Atlantic has been scarce in the last decades, except for the recent studies conducted on South African coasts (Landschoff & Rahayu, 2018). After a golden period of studies on African coasts leaded by J. Forest among others in the mid‐19th to 20th centuries (see Barnard, 1950, 1955; Chevreux & Bouvier, 1892; Forest, 1952, 1961; Kensley, 1981), where most of the current accepted species were described, the account of *Diogenes* species occurring in this vast area has remained almost inalterable, in part due to the consideration of *D*. *pugilator* as a widespread species with high morphological variability (Forest, 1955; McLaughlin et al., 2010).

In a previous work, the examination of the variability of *Diogenes* species inhabiting a relatively small area as the Iberian Peninsula showed that there were in fact several different species formally assigned to *D*. *pugilator*, leading to the description of a new species (*D*. *armatus*), the resurrection of an ancient synonymy (*D*. *curvimanus*) and the redescription of *D*. *pugilator sensu* Roux, 1829 (Almón et al., 2021). The redescription of the original species, as well as the designation of neotypes that allow comparison with other specimens, marks a reference point that did not exist until that moment (since the specimens deposited by Roux seem to have been lost), thus providing a stable reference from which to build future studies. The application of an integrative taxonomy approach provides a great opportunity to tackle many ancient conflicts from a new and comprehensive perspective. The species described here also belong to the so‐called *D*. *pugilator* species complex and adds two new species for the genus and for the Iberian Peninsula carcinofauna. Based on the current knowledge about their distribution, their geographical origin is not clear. Although they are present in the southern part of the Iberian Peninsula, the molecular results suggest a closer relationship with other African species than with European ones, which opens the possibility of a recent arrival to Europe from northern African coasts, where they are also present. Future studies will contribute to clarify the actual distribution range of the new species, probably revealing along the way, new species still to be discovered.

The revision of the *Diogenes* species present along European and African coasts is a pending subject that will require cooperative work to overreach the great task that imply the study of this vast and complex area, but at the same time it represents an interesting challenge that will probably yield discoveries that will drastically change the composition and general knowledge about the genus as a whole.

## CONFLICTS OF INTEREST

The authors declare no conflicts of interest.

## AUTHOR CONTRIBUTION

**Bruno Almón**: Conceptualization (equal), data curation (equal), formal analysis (lead), funding acquisition (equal), investigation (equal), methodology (equal), project administration (equal), resources (equal), software (equal), validation (equal), visualization (equal), writing—original draft (lead), writing—review and editing (equal). **Jose A. Cuesta**: Conceptualization (equal), data curation (equal), formal analysis (equal), funding acquisition (lead), investigation (equal), methodology (equal), project administration (equal), resources (equal), software (equal), supervision (lead), validation (equal), visualization (lead), writing—original draft (equal), writing—review and editing (equal). **J. Enrique García‐Raso**: Conceptualization (equal), data curation (equal), formal analysis (equal), funding acquisition (lead), investigation (equal), methodology (equal), project administration (equal), resources (lead), software (equal), supervision (lead), validation (equal), visualization (equal), writing—original draft (equal), writing—review and editing (equal).

## Supporting information

Figure S1

Figure S2

Figure S3

## Data Availability

The data underlying this article are available in the article and in its online supplementary material. DNA sequences and related data are publicly available on the National Center for Biotechnology public databases (https://www.ncbi.nlm.nih.gov/). The data associated with each of the specimens examined are included in the text, in the appropriate sections. Accession number for sequences downloaded from public databases is included in Table 2, along with those generated for this project.

## References

[ece38844-bib-0001] Almón, B. , Cuesta, J. A. , Schubart, C. D. , Armenia, L. , & García‐Raso, J. E. (2021). Redescription of the hermit crab *Diogenes pugilator* (Decapoda: Anomura) reveals the existence of a species complex in the Atlanto‐Mediterranean transition zone, resulting in the resurrection of *D. curvimanus* and the description of a new species. Zoological Journal of the Linnean Society, zlab093, 1–31. 10.1093/zoolinnean/zlab093

[ece38844-bib-0002] Asakura, A. (2006). Shallow water hermit crabs of the families Pylochelidae, Diogenidae, Paguridae (Crustacea: Decapoda: Anomura) from the Sea of Japan, with a description of a new species of *Diogenes* . Bulletin of the Toyana Science Museum, 29, 23–103.

[ece38844-bib-0003] Asakura, A. (2020). Hermit crabs of the genus *Diogenes* Dana, 1851 (Crustacea: Decapoda: Diogenidae) collected during the Albatross Philippine Expedition, 1907–1910, including descriptions of three new species. Publications of the Seto Marine Biological Laboratory, 45, 1–46.

[ece38844-bib-0004] Asakura, A. , & Godwin, S. (2006). *Diogenes patae* n. sp., a new species of hermit crab (Crustacea: Decapoda: Anomura: Diogenidae) from American Samoa. Zoosystema, 28(2), 457–463.

[ece38844-bib-0005] Asakura, A. , & Tachikawa, H. (2010). *Diogenes holthuisi*, a new species of hermit crab (Decapoda, Anomura, Diogenidae) from shallow waters of the Ogasawara (Bonin) Islands, Japan. In C. H. J. M. Fransen , S. De Grave , & P. K. L. Ng (Eds.), Studies on Malacostraca: Lipke Bijdeley Holthuis Memorial Volume. *Crustaceana Monographs*, Brill (pp. 133–144).

[ece38844-bib-0006] Balss, H. (1921). Crustacea VI: Decapoda Anomura (Paguridae) und Brachyura (Dromiacea bis Brachygnatha. In W. Michaelsen (Ed.), Beiträge zur Kenntnis der Meeresfauna Westafrikas. Band III, Lieferung 2. L. Friederichsen & Co. (pp. 37–67).

[ece38844-bib-0007] Barnard, K. H. (1950). Descriptive catalogue of South African decapod Crustacea (crabs and shrimps). Annals of the South African Museum, 38, 1–837.

[ece38844-bib-0008] Barnard, K. H. (1955). Additions to the fauna‐list of South African Crustacea and Pycnogonida. Annals of the South African Museum, 43, 1–107.

[ece38844-bib-0009] Canning‐Clode, J. , & Carlton, J. C. (2017). Refining and expanding global climate change scenarios in the sea: Poleward creep complexities, range termini, and setbacks and surges. Diversity and Distributions, 23, 463–473. 10.1111/ddi.12551

[ece38844-bib-0010] Chevreux, E. , & Bouvier, E. L. (1892). Voyage de la goèlette Melita aux Canaries et au Sénégal. Note préliminaire sur les paguriens. Bulletin De La Société Zoologique De France, 16, 252–256.

[ece38844-bib-0101] Clément, M. C. (1874). Description d’un pagure nouveau. Bulletin De La Société D’etude Des Sciences Naturelles De Nimes, 2, 155–157.

[ece38844-bib-0011] Crandall, K. A. , & Fitzpatrick, J. E. Jr (1996). Crayfish molecular systematics: using a combination of procedures to estimate phylogeny. Systematic Biology, 45, 1–26. 10.1093/sysbio/45.1.1

[ece38844-bib-0012] Cuesta, J. A. , Almón, B. , Pérez‐Dieste, J. , Trigo, J. E. , & Bañón, R. (2016). Role of Ships' hull fouling and tropicalization process on European carcinofauna: new records in Galician waters (NW Spain)". Biological Invasions, 18(3), 619–630. 10.1007/s10530-015-1034-9

[ece38844-bib-0013] Edgar, R. C. (2004). MUSCLE: multiple sequence alignment with high accuracy and high throughput. Nucleic Acids Research, 32(5), 1792–1797. 15034147 10.1093/nar/gkh340 PMC390337

[ece38844-bib-0014] Encarnação, J. , Morais, P. , Baptista, V. , Cruz, J. , & Teodósio, M. A. (2019). New Evidence of Marine Fauna Tropicalization off the Southwestern Iberian Peninsula (Southwest Europe). Diversity, 11, 48. 10.3390/d11040048

[ece38844-bib-0015] Estoup, A. , Largiadèr, C. R. , Perrot, E. , & Chourrout, D. (1996). Rapid one tube DNA extraction for reliable PCR detection of fish polymorphic marker and transgenes. Molecular Marine Biology and Biotechnology, 5, 295–298.

[ece38844-bib-0016] Forest, J. (1952). Remarques sur les genres *Diogenes* Dana et *Troglopagurus* Henderson à propos de la description d’un Paguridae nouveau de la côte occidentale d’Afrique, *Diogenes mercatoris* sp. nov. Bulletin De L’institut Royal Des Sciences Naturelle De Belgique, 28, 1–15.

[ece38844-bib-0017] Forest, J. (1955). Crustacés Décapodes, Pagurides. Expédition océanographique Belge dans les eaux côtières africaines de l’Atlantique Sud (1948–1949). Résultats Scientifiques, 3, 23–147.

[ece38844-bib-0018] Forest, J. (1961). Pagurides de l’Afrique occidentale. Atlantide Reports, 6, 203–250.

[ece38844-bib-0019] García‐Raso, J. E. (1985). Presencia de una población de *Brachynotus atlanticus* Forest, 1957 (Crustacea, Decapoda, Brachyura: Grapsidae) en el sur de la Península Ibérica. Boletim Da Sociedade Portuguesa De Entomologia, Supl, 19–26.

[ece38844-bib-0020] García‐Raso, J. E. (1993). New record of other African species of Crustacea Decapoda, *Cycloes cristata* (Brulle), from European and Mediterranean waters. Bios, 1(1), 215–221.

[ece38844-bib-0021] García‐Raso, J. E. , & Manjón‐Cabeza, E. (1996). New Record of *Liocarcinus mcleayi* (Barnard, 1947), New Combination (Decapoda, Brachyura, Portunidae) from South Europe. Crustaceana, 69(1), 84–93.

[ece38844-bib-0022] García‐Raso, J. E. , Salmerón, F. , Baro, J. , Marina, P. , & Abelló, P. (2014). The tropical African hermit crab *Pagurus mbizi* (Decapoda, Paguridae) with an established population in the Western Mediterranean Sea: a new alien species or filling gaps in the knowledge of the distributions? Mediterranean Marine Science, 15(1), 172–178.

[ece38844-bib-0023] Gibert, J. M. , Muñiz, F. , Belaústegui, Z. , & Hyžný, M. (2013). Fossil and Modern Fiddler Crabs (*Uca tangeri*: Ocypodidae) and Their Burrows from SW Spain: Ichnologic and Biogeographic Implications. Journal of Crustacean Biology, 33(4), 537–551. 10.1163/1937240X-00002151

[ece38844-bib-0024] González‐Ortegón, E. , García‐Raso, J. E. , Calado, R. , López de la Rosa, I. , Guerrero, M. , & Cuesta, J. A. (2020). Atlantic Expansion of the African Caridean Shrimp *Lysmata uncicornis* Holthuis & Maurin, 1952 (Caridea: Lysmatidae). Marine Biodiversity, 50(2), 26. 10.1007/s12526-020-01056-w

[ece38844-bib-0025] González‐Ortegón, E. , Jenkins, S. , Galil, B. S. , Drake, P. , & Cuesta, J. A. (2020). Accelerated invasion of decapod crustaceans in the southernmost point of the Atlantic coast of Europe: A non‐natives’ hot spot? Biological Invasions, 22, 3487–3492. 10.1007/s10530-020-02345-y

[ece38844-bib-0026] Guerrero, E. , Abelló, P. , Lombarte, A. , Villanueva, R. , Ramón, M. , Sabatés, A. , & Santos, R. (2020). Biological Reference Collections ICM‐CSIC. v1.28. Institute of Marine Sciences (ICM‐CSIC). Dataset/Occurrence. 10.15470/qlqqdx

[ece38844-bib-0027] Hall, T. (1999). BioEdit: a user‐friendly biological sequence alignment editor and analysis program for Windows 95/98/NT. Nucleic Acids Symposium Series, 41, 95–98.

[ece38844-bib-0102] Herbst, J. F. W. (1791–1796). Versuch einer Naturgeschichte der Krabben und Krebse nebst einer systematischen Beschreibung ihrer verschiedenen Arten. Zweyter Band. Mit XXV Kupfer‐Tafeln und Register. Krebse. Gottlieb August Lange, Berlin und Stralsund, viii + 225 + [1] pp. 25 pls.

[ece38844-bib-0028] Holthuis, L. B. (1977). First record of the family Ogyrididae from European waters (Decapoda, Caridea). Crustaceana, 33, 108–111. 10.1163/156854077X00340

[ece38844-bib-0029] Igawa, M. , & Kato, M. (2017). A new species of hermit crab, *Diogenes heteropsammicola* (Crustacea, Decapoda, Anomura, Diogenidae), replaces a mutualistic sipunculan in a walking coral symbiosis. PLoS One, 12(9), e0184311. 28931020 10.1371/journal.pone.0184311 PMC5606932

[ece38844-bib-0030] Kensley, B. (1981). On the zoogeography of southern African decapod Crustacea, with a distributional checklist of the species. Smithsonian Contributions to Zoology, 338, 1–64.

[ece38844-bib-0031] Komai, T. , Liang, J. , & Yang, T. (2012). Records of four species of the shallow water hermit crab genus *Diogenes* (Crustacea: Decapoda: Anomura: Diogenidae) from southern China, with description of a new species. Journal of Natural History, 46(19–20), 1219–1248.

[ece38844-bib-0032] Komai, T. , Ravinesh, R. , & Kumar, A. B. (2018). A new species of the hermit crab genus *Diogenes* Dana, 1851 (Decapoda: Anomura: Diogenidae) from southern India. Zootaxa, 4504(2), 243–252. 10.11646/zootaxa.4504.2.5 30486027

[ece38844-bib-0033] Komai, T. , Reshmi, R. , & Kumar, A. B. (2013). A new species of the hermit crab genus *Diogenes* (Crustacea: Decapoda: Anomura: Diogenidae) from southern India. Zootaxa, 3613(4), 380–390. 24698925 10.11646/zootaxa.3613.4.5

[ece38844-bib-0034] Komai, T. , & Yoshida, R. (2020). A new species of the hermit crab genus *Diogenes* Dana, 1851 (Decapoda: Anomura: Diogenidae) from shallow coastal waters in Japan. Zootaxa, 4722, 571–582. 10.11646/zootaxa.4722.6.4 32230600

[ece38844-bib-0035] Kumar, S. , Stecher, G. , Li, M. , Knyaz, C. , & Tamura, K. (2018). MEGA X: Molecular Evolutionary Genetics Analysis across computing platforms. Molecular Biology and Evolution, 35, 1547–1549. 10.1093/molbev/msy096 29722887 PMC5967553

[ece38844-bib-0036] Landschoff, J. , & Rahayu, D. L. (2018). A new species of the hermit crab genus *Diogenes* (Crustacea: Decapoda: Diogenidae) from the coast of KwaZulu‐Natal, South Africa. Zootaxa, 4379(2), 268–278. 10.11646/zootaxa.4379.2.7 29689988

[ece38844-bib-0037] Lemaitre, R. , & McLaughlin, P. (2022). World Paguroidea & Lomisoidea Database. *Diogenes* Dana, 1851. Accessed through: World Register of Marine Species at: https://www.marinespecies.org/aphia.php?p=taxdetails&id=106843 on 2022‐02‐09

[ece38844-bib-0104] Linnaeus, C. (1767). Systema naturae per regna tria naturae: Secundum classes, ordines, genera, species, cum characteribus, differentiis, synonymis, locis. Ed. 12. 1., Regnum Animale. 1 & 2. Holmiae [Stockholm], Laurentii Salvii. (pp. 1–532 [1766] pp. 533–1327 [1767]).

[ece38844-bib-0038] McLaughlin, P. A. , & Clark, P. F. (1997). A review of the *Diogenes* (Crustacea, Paguroidea) hermit crabs collected by Bedford and Lanchester from Singapore, and from the ‘Skeat’ Expedition to the Malay Peninsula, with a description of a new species and notes on *Diogenes intermedius* De Man, 1892. Bulletin of the Natural History Museum, London, (Zoology) 63: 33–49.

[ece38844-bib-0039] McLaughlin, P. A. , & Holthuis, L. B. (2001). In pursuit of J. F. W. Herbst's species of *Diogenes* (anomura: Paguroidea: Diogenidae). Journal of Crustacean Biology, 21, 257–273.

[ece38844-bib-0040] McLaughlin, P. A. , Komai, T. , Lemaitre, R. , & Rahayu, D. L. (2010). Annotated checklist of anomuran decapod crustaceans of the world (exclusive of the Kiwaoidea and families Chirostylidae and Galatheidae of the Galatheoidea) Part I – Lithodoidea, Lomisoidea and Paguroidea. Raffles Bulletin of Zoology, 23, 107.

[ece38844-bib-0041] Mock, E. , & Schubart, C. D. (2021). Reconstruction of intrageneric relationships within the Indo‐West Pacific littoral crab genus *Metopograpsus* (Decapoda: Brachyura: Grapsidae): an alternative speciation order according to a 28S rDNA molecular phylogeny. Crustaceana, 94(11‐12), 1313–1325. 10.1163/15685403-bja10156

[ece38844-bib-0042] Morgan, G. J. , & Forest, J. (1991). Seven new species of hermit crabs from Northern and Western Australia (Decapoda, Anomura, Diogenidae). Bulletin Du Muséum National D'histoire Naturelle, Paris, 4(12), 649–689.

[ece38844-bib-0043] Muñoz, E. , & García‐Isarch, E. (2021). Colección de Crustáceos Decápodos y Estomatópodos del Centro Oceanográfico de Cádiz (CCDE‐IEOCD). v1.20. Oceanographic Center of Cadiz, Spanish Institute of Oceanography (IEO). Dataset/Occurrence. https://ipt.gbif.es/resource?r=ccde‐ieocd&v=1.20

[ece38844-bib-0044] Palumbi, S. , Martin, A. , Romano, S. , McMillan, W. O. , Stice, L. , & Grabowski, G. (1991). The simple fool’s guide to PCR, Ver. 2.0 Honolulu, University of Hawaii.

[ece38844-bib-0045] Patterson, C. , Laing, C. , & Early, R. (2022). The range expansion of *Clibanarius erythropus* to the UK suggests that other range‐shifting intertidal species may not follow. Marine Biology, 169, 10.1007/s00227-021-04008-5

[ece38844-bib-0046] Pérez‐Miguel, M. , Drake, P. , García‐Raso, J. E. , Maman‐Menéndez, L. , Navas, J. I. , & Cuesta, J. A. (2019). European Pinnotheridae (Crustacea, Decapoda, Brachyura): Species, Distribution, Host use and DNA barcodes. Marine Biodiversity, 49(1), 57–68. 10.1007/s12526-017-0754-8

[ece38844-bib-0047] Posada, D. , & Buckley, T. R. (2004). Model selection and model averaging in phylogenetics: Advantages of Akaike information criterion and Bayesian approaches over likelihood ratio tests. Systematic Biology, 53(5), 793–808. 10.1080/10635150490522304 15545256

[ece38844-bib-0048] Pozuelo, M. , Arias, A. , Rodríguez, A. , & Pettenghi, J. (1976). Presencia de *Scyllarus posteli* Forest en la Bahía de Cádiz (región sudatlántica española). Investigación Pesquera, 40, 85–93.

[ece38844-bib-0049] Rahayu, D. L. (1996). Notes on littoral hermit crabs (excluding Coenobitidae) (Crustacea: Decapoda: Anomura) mainly from Singapore and Peninsular Malaysia. Raffles Bulletin of Zoology, 44(2), 335–355.

[ece38844-bib-0050] Rahayu, D. L. (2012). A new species of the hermit crab genus *Diogenes* Dana, 1851 (Decapoda, Anomura, Diogenidae) from Lombok, Indonesia. Studies on Eumalacostraca: a Homage to Masatsune Takeda, 263–274.

[ece38844-bib-0051] Rahayu, D. L. (2015). New record and new species of the hermit crab genus *Diogenes* Dana, 1851 (Decapoda: Anomura: Diogenidae) from Singapore. Raffles Bulletin of Zoology, 31, 182–192.

[ece38844-bib-0052] Rahayu, D. L. , & Forest, J. (1995). Le genre *Diogenes* (Crustacea, Decapoda, Diogenidae) en Indonésie, avec la description de six espèces nouvelles. Bulletin Du Muséum National D'histoire Naturelle, Paris, 16(2–4), 383–415.

[ece38844-bib-0053] Rahayu, D. L. , & Hortle, K. G. (2002). The genus *Diogenes* (Decapoda, Anomura, Diogenidae) from Irian Jaya, Indonesia with description of a new species. Crustaceana, 75(3–4), 609–619.

[ece38844-bib-0054] Rossignol, M. (1957). Crustacés Décapodes marins de la région de Pointe‐Noire. In J. Cowgnon , M. R. Rossignol , & C. Ch. Mollusques (Eds.), Crustacés, Poissons marins des côtes d'A.E.F. en collection au Centre d'Océanographie de l'I.E.C (pp. 71–136). ORSTOM.

[ece38844-bib-0055] Rossignol, M. (1962). Note sur le genre *Diogenes* Dana 1851 (crustacés décapodes anomoures fam. *Paguridae*). In B. Jacques & R. Robert (Eds.), Travaux du Centre Océanographique de Pointe‐Noire: travaux du Laboratoire d'Océanographie Biologique: études diverses (pp. 147–153). ORSTOM. multigr.

[ece38844-bib-0100] Roux, P. (1828–1830). Crustacés de la Méditerranée et de son littoral, décrits et lithographiés. Éditions Levrault. [176 unnumbered pp., 45 pls; published in 9 parts: 1, 2 (pls 1–10), 1828; 3 (pls 11–15), 1829; 4–9 (pls 16–45) 1830].

[ece38844-bib-0056] Schubart, C. D. , Cuesta, J. A. , & Felder, D. L. (2002). Glyptograpsidae, a new brachyuran family from Central America: larval and adult morphology, and a molecular phylogeny of the Grapsoidea. Journal of Crustacean Biology, 22, 28–44.

[ece38844-bib-0057] Schubart, C. D. , Diesel, R. , & Hedges, S. B. (1998). Rapid evolution to terrestrial life in Jamaican crabs. Nature, 393, 363–365.

[ece38844-bib-0058] Schubart, C. D. , & Huber, M. G. J. (2006). Genetic comparisons of German populations of the stone crayfish, *Austropotamobius torrentium* (Crustacea: Astacidae). Bulletin Français De La Pêche Et De La Pisciculture, 380–381, 1019–1028.

[ece38844-bib-0059] Siddiqui, F. A. , & McLaughlin, P. A. (2003). A new species of the hermit crab genus *Diogenes* (Decapoda: Anomura: Paguroidea: Diogenidae) from Pakistan, with a comparative diagnosis of *D. guttatus* Henderson, 1888. Proceedings of the Biological Society of Washington, 116(4): 956–966.

[ece38844-bib-0060] Talavera, G. , & Castresana, J. (2007). Improvement of phylogenies after removing divergent and ambiguously aligned blocks from protein sequence alignments. Systematic Biology, 56, 564–577. 10.1080/10635150701472164 17654362

[ece38844-bib-0061] Trivedi, J. N. , Osawa, M. , & Vachhrajani, K. D. (2016). A new species of the genus *Diogenes* Dana, 1851 (Crustacea: Decapoda: Anomura: Diogenidae) from Gujarat, northwestern India. Zootaxa, 4208(2), 189. 10.11646/zootaxa.4208.2.6 27988534

[ece38844-bib-0062] Xiao, L. C. , Sha, Z. L. , & Wang, Y. L. (2015). A new species of the genus *Diogenes* (Decapoda, Anomura, Diogenidae) from the South China Sea. Crustaceana, 88(12–14), 1439–1445.

