# Supplementary figures and images for: Two new hermit crab species of Diogenes (Crustacea: Decapoda: Diogenidae) from Atlanto‐Mediterranean coasts of Iberian Peninsula: Poleward migrants or merely overlooked indigenous species?
Source: Ecol Evol. 2022 May 19;12(5):e8844. doi: 10.1002/ece3.8844 (PMC9120568; doi:10.1002/ece3.8844)

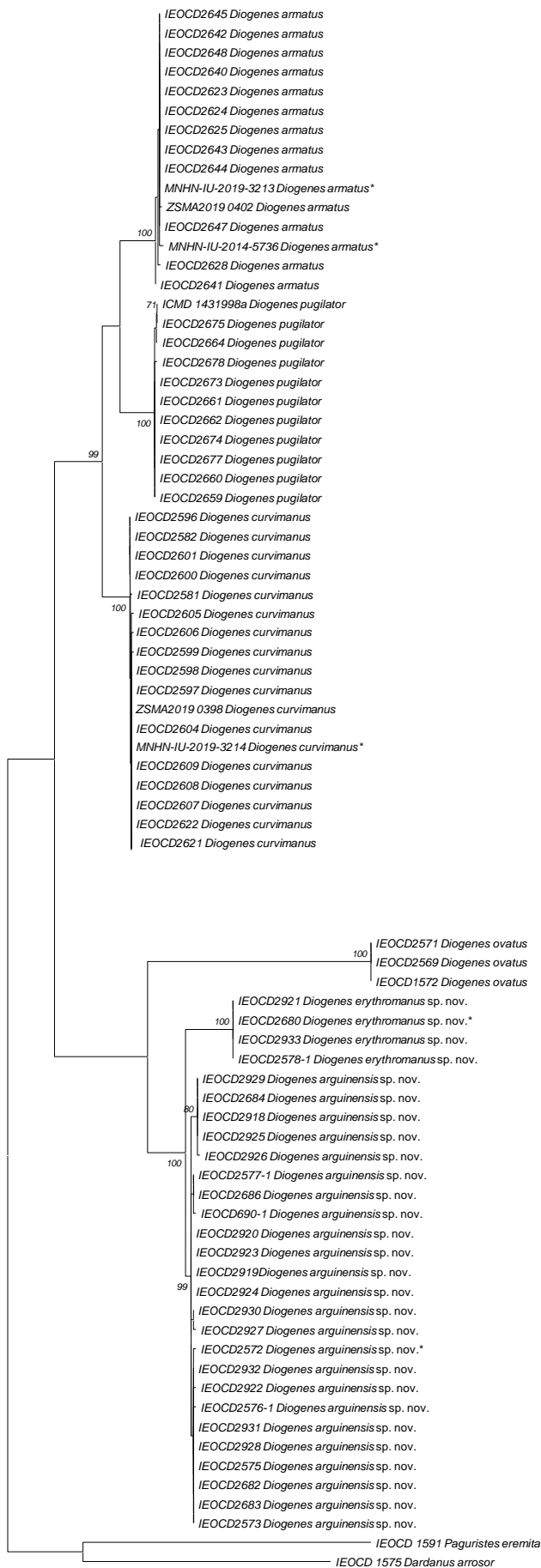

Supplement: Supplementary file 1 — Figure S1 [file ECE3-12-e8844-s003.pdf]

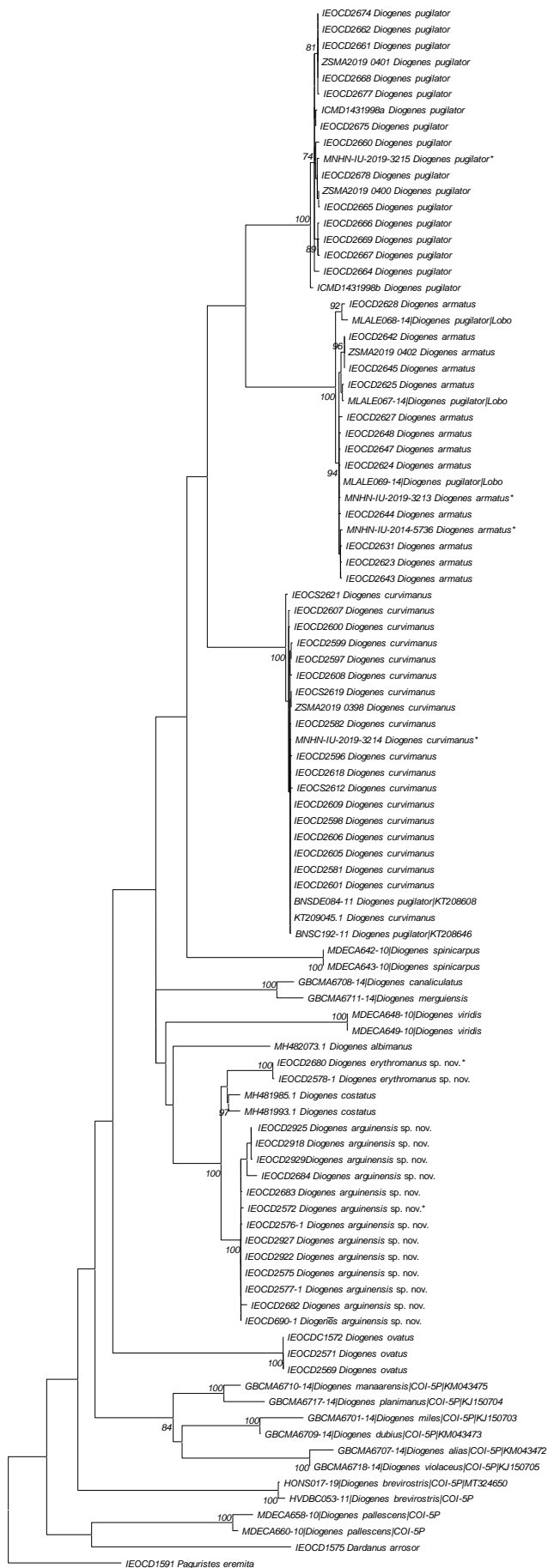

Supplement: Supplementary file 2 — Figure S2 [file ECE3-12-e8844-s002.pdf]

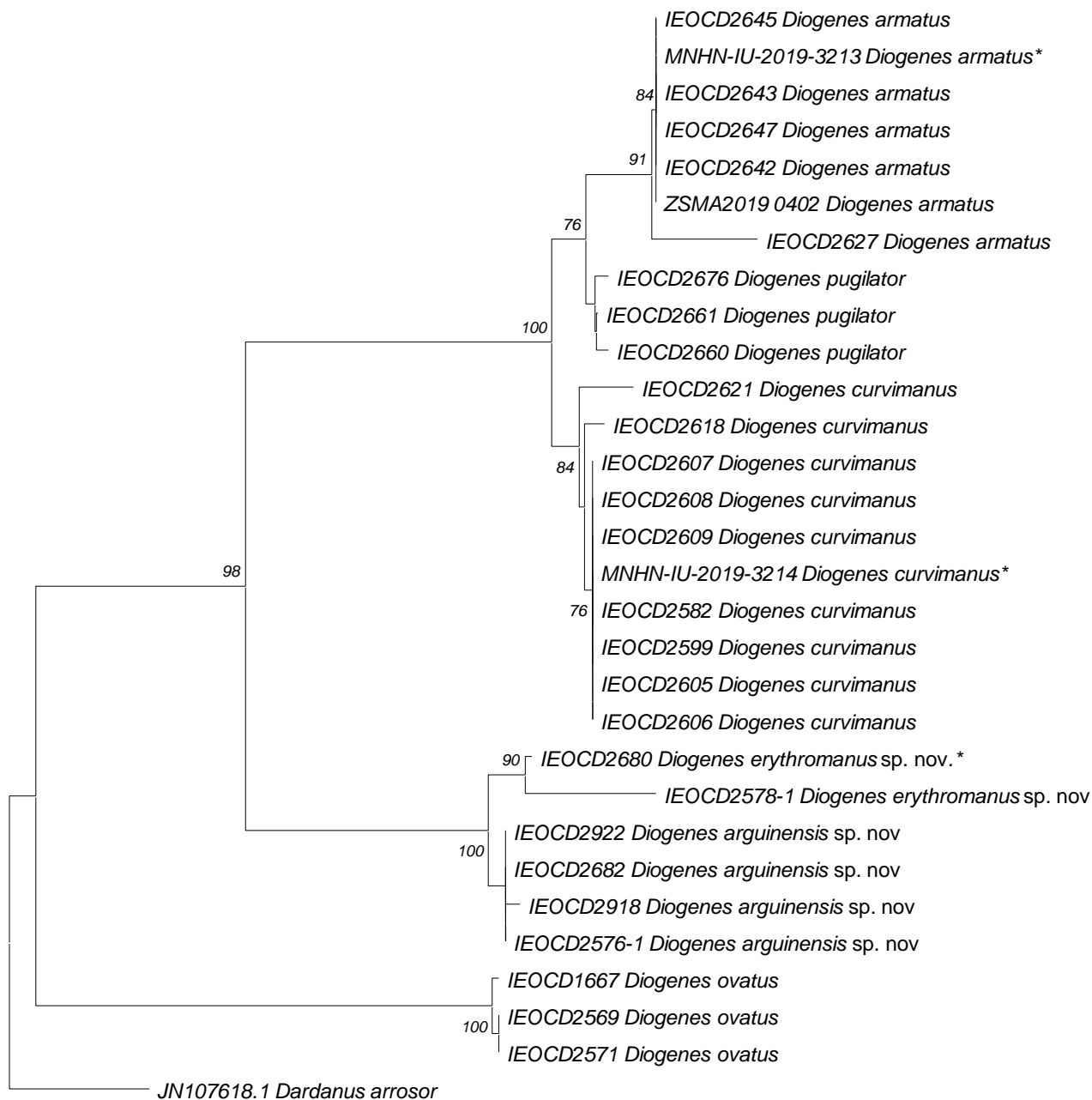

0.01

Supplement: Supplementary file 3 — Figure S3 [file ECE3-12-e8844-s001.pdf]
